# Engineered packaging cell line for the enhanced production of baboon-enveloped retroviral vectors

**DOI:** 10.1016/j.omtn.2024.102389

**Published:** 2024-11-13

**Authors:** Denise Klatt, Lucia Sereni, Boya Liu, Pietro Genovese, Axel Schambach, Els Verhoeyen, David A. Williams, Christian Brendel

**Affiliations:** 1 Gene Therapy Program, Dana Farber/Boston Children’s Cancer and Blood Disorders Center, Harvard Medical School, Boston, MA 02115, USA; 2 Division of Hematology/Oncology, Boston Children’s Hospital, Harvard Medical School, Boston, MA 02115, USA; 3 Institute of Experimental Hematology, Hannover Medical School, 30625 Hannover, Germany; 4 Centre International de Recherche en Infectiologie (CIRI), Université Lyon, Université Claude Bernard Lyon 1, INSERM, U1111, CNRS, UMR 5308, Ecole Normale Supérieure de Lyon, 69007 Lyon, France; 5 Université Côte d'Azur, INSERM U1065, Centre Méditerranéen de Médecine Moléculaire, 06200 Nice, France

**Keywords:** MT: Delivery Strategies, packaging cell line, BaEVRLess envelope, ASCT2 knockout, α-retroviral vectors, CD47 overexpression, β2-microglobulin knockout, HSC gene therapy, retroviral vector production

## Abstract

The baboon endogenous retrovirus (BaEV) glycoprotein is superior to the commonly used vesicular stomatitis virus glycoprotein (VSVg) for retroviral gene transfer into resting hematopoietic stem cells and lymphocyte populations. The derivative BaEVRLess (lacking the R domain) produces higher viral titers compared with wild-type BaEV, but vector production is impaired by syncytia formation and cell death of the HEK293T cells due to the high fusogenic activity of the glycoprotein. This lowers viral titers, leads to increased batch-to-batch variability, and impedes the establishment of stable packaging cell lines essential for the economical production of viral supernatants. Here, we show that knockout of the entry receptor ASCT2 in HEK293T producer cells eliminates syncytia formation, resulting in a 2-fold increase in viral titers, reduced toxicity of viral supernatants, and enables the generation of stable packaging cell lines. In successive steps, we stably integrated BaEVRLess and α-retroviral a.Gag/Pol expression cassettes and isolated clones supporting titers up to 10^8^ to 10^9^ infectious particles/mL, a 10-fold increase in concentrated viral titers. The additional overexpression of CD47 and knockout of β2-microglobulin in the packaging cell line are tailored for future use in *in vivo* gene therapy applications by reducing non-specific uptake by macrophages and the immunogenicity of viral particles.

## Graphical abstract

**Figure:**
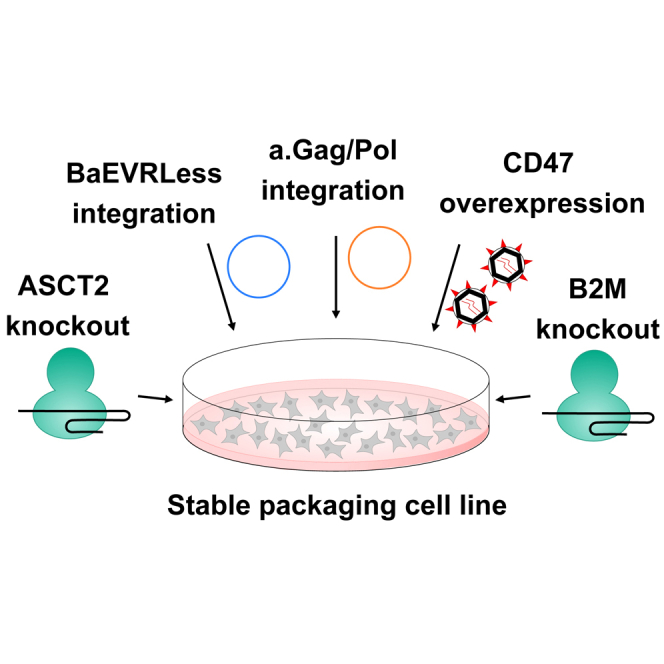

## Teaser

KO of the ASCT2 entry receptor in 293T improves titers and virus quality of BaEVRless pseudotyped retroviral vectors. This enables the generation of stable packaging cells through sequential introduction of α-retroviral vector components. Overexpression of CD47 and B2M KO are intended to improve performance of the virus for *in vivo* administration.

## Introduction

Retroviral vector-mediated hematopoietic stem cell (HSC) gene therapy has proven efficacious for the treatment of various genetic diseases, such as primary immunodeficiencies or sickle cell anemia.^1^^,^^2^^,^^3^ Similarly, CAR-T cell-based therapies rely on *ex vivo* retroviral gene transfer. The most utilized delivery system is lentiviral (LV) vectors pseudotyped with the vesicular stomatitis virus glycoprotein (VSVg), which enter cells by binding to the broadly expressed low-density lipoprotein receptor (LDL-R). However, the LDL-R is expressed at very low levels on quiescent HSCs, and unstimulated T and B cells, which results in poor transduction rates.^4^ To transduce HSCs efficiently *ex vivo,* they must be activated by a cocktail of stimulating cytokines during multi-day *ex vivo* culture, which reduces HSC repopulation potential.^5^^,^^6^^,^^7^ Alternative viral envelopes derived from the baboon endogenous retrovirus glycoprotein (BaEV) or the feline endogenous retrovirus (RD114) have been shown to facilitate gene transfer also into quiescent human HSCs,^8^ which could enable shortened *ex vivo* culture time under minimally stimulating conditions to retain optimal HSC repopulation potential or even be adapted for *in vivo* gene transfer into quiescent HSCs.^9^^,^^10^^,^^11^ The entry receptors for BaEV variants and RD114 are the neutral amino acid transporters ASCT1 and ASCT2 or ASCT2 only, respectively. These receptors are highly expressed on resting HSCs, and superior gene transfer has also been reported for T, B, and NK cells, where viral vectors pseudotyped with BaEV envelope variants outperform other envelopes including VSVg.^12^^,^^13^^,^^14^^,^^15^^,^^16^

In this study, we focus on the BaEVRLess variant, a derivative of BaEV created by the deletion of the fusion-inhibitory R domain. This variant achieves 3-log higher titers than the wild-type (WT) BaEV glycoprotein.^8^ However, as an undesired side effect, deletion of the R domain results in a highly fusogenic glycoprotein. Thus, expression of BaEVRLess during vector production causes the fusion of HEK293T vector producer cells and the formation of giant syncytia, leading to cell detachment and cell death and precluding continuous viral vector production.^17^^,^^18^

We hypothesized that knockout (KO) of the entry receptors would suppress BaEVRLess-mediated cell fusions, overcome this critical barrier to attain higher titers and enable the generation of a stable production system. Continuous stable viral producer cell lines are important for the transition from single batch transient transfection manufacturing systems to scalable manufacturing processes, which are a prerequisite for cost reduction, elimination of batch-related variability, and ultimately for more widespread use of gene therapy.^19^ However, permanent expression of LV components, such as the HIV-1 protease or the VSVg envelope, can be cytotoxic to the producer cells.^20^ Thus, stable LV producer cell lines are not commonly used. α-Retroviral (αRV) vectors, which are derived from a closely related genus, can also transduce non-dividing cells and, in contrast to LV vectors, can be efficiently produced in stable cell lines.^21^^,^^22^^,^^23^^,^^24^

An additional advantage of BaEV-pseudotyped αRV particles is their potential for *in vivo* applications. While *in vivo* delivery is in its infancy, it carries great potential for many diseases. BaEV-pseudotyped particles, unlike VSVg-pseudotyped retroviral particles, are not neutralized by exposure to human serum complement and thus are a better potential candidate for *in vivo* delivery into cells expressing high levels of ASCT1 or ASCT2, such as HSCs.^8^^,^^25^^,^^26^^,^^27^ *In vivo* application of viral vectors is furthermore hampered by clearance of viral particles via the immune system, for example uptake of viral particles by phagocytes, and ensuing innate immune responses.^28^^,^^29^ Non-specific uptake of injected viral particles by macrophages can be suppressed by overexpression of CD47 on LV particles.^28^^,^^30^ This molecule binds to the checkpoint molecule SIRPA expressed on macrophages and provides a “don’t eat me” signal, thereby reducing macrophage-mediated clearance and innate immune responses.^31^ To further reduce the immunogenicity of the viral particles, MHC-I-free particles can be generated from a β2-microglobulin (B2M) KO packaging cell line as shown by Milani et al.^29^

The objective of this work was to overcome current problems and limitations associated with the production of BaEVRLess-pseudotyped retroviral vectors, aiming for enhanced viral titers and quality, while also facilitating the establishment of stable packaging cell lines for consistent and cost-effective vector production. ASCT2 KO suppressed cell fusion, and a stable BaEVRLess αRV packaging cell line was realized by sequential integration of the BaEVRLess envelope and αRV structural components into HEK293T cells for provision of these proteins in *trans* for vector packaging. In the next step, we aimed to enhance the performance of viral particles for potential *in vivo* gene transfer by overexpression of CD47, which reduced the non-specific uptake of viral particles by macrophages via engagement of the SIRPA receptor, and B2M KO to generate MHC-I-free viral particles with reduced immunogenicity. In summary, we show that BaEVRLess entry receptor KO increases titers and enables the generation of a stable packaging cell line for αRV vectors, which additionally overexpress CD47 and lack MHC-I for future *in vivo* gene transfer.

## Results

### KO of ASCT1 and ASCT2 in HEK293T cells

**Figure 1:**
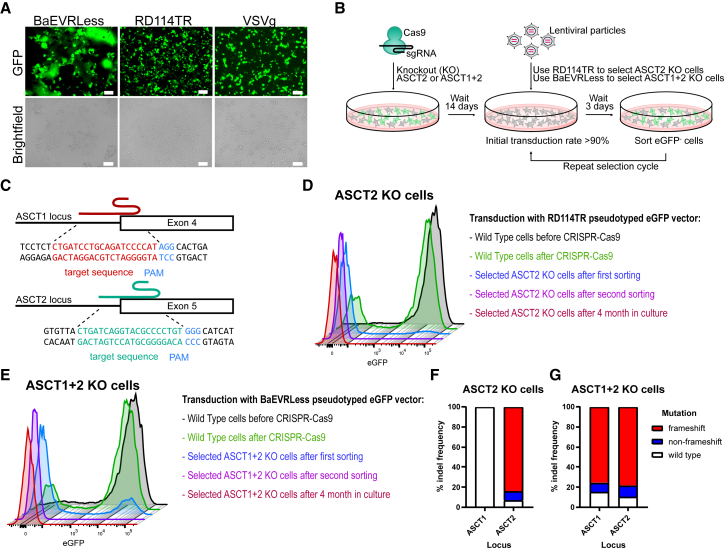
Knockout of ASCT1 and ASCT2 in HEK293T cells (A) Microscopic pictures of HEK293T cells during vector production. Vector production of BaEVRLess-pseudotyped viral particles (left), RD114TR-pseudotyped viral particles (middle), and VSVg-pseudotyped viral particles (right). Scale bars, 100 μm. (B) Schematic showing generation of ASCT2 and ASCT1+2 KO cell lines using CRISPR-Cas9. (C) Schematic indicating the sgRNA binding sites and Cas9 cleavage sites in the ASCT1 and ASCT2 locus. (D and E) Enrichment of ASCT2 KO (D) and ASCT1+2 (E) via two rounds of transduction using an eGFP vector and cell sorting of eGFP-negative cells. (F and G) Distribution of editing outcomes at each target site within the ASCT1 and ASCT2 locus in ASCT2 KO cells (F) and in ASCT1+2 KO cells (G).

The BaEVRLess envelope is highly fusogenic, leading to increased syncytia formation during vector production compared with other viral envelopes, such as VSVg or RD114TR (Figure 1A). While RD114TR and BaEVRLess both bind to ASCT2, BaEV-derived glycoproteins additionally utilize ASCT1 as entry receptor. To eliminate cell fusion and to reduce the risk of replication-competent retroviral (RCR) particle formation via superinfection in the producer cells, we used CRISPR-Cas9 to knock out ASCT2 alone or in combination with ASCT1 in HEK293T cells (Figure 1B). The sgRNAs were designed to target exon 4 of the ASCT1 locus and exon 5 of the ASCT2 locus (Figure 1C). The resulting ASCT2 KO or the ASCT1 and ASCT2 (ASCT1+2) double KO cells were selected based on their resistance to infection with either eGFP encoding RD114TR-pseudotyped LV vectors for ASCT2 KO cells or BaEVRLess-pseudotyped vectors for ASCT1+2 KO cells. The KO cells were enriched in two consecutive rounds via sorting of eGFP-negative cells. At the end of the selection, KO cells yielded <1% eGFP+ cells after transduction with the respective viral pseudotype (Figures 1D, 1E, S1A, and S1B) and revealed complete loss of the ASCT2 protein as confirmed by western blot (Figure S1C). To verify gene disruption at the ASCT1 and ASCT2 loci, genomic DNA was isolated and subjected to Sanger sequencing of the respective genomic location. Sequencing analysis of ASCT2-edited cells revealed 84.4% frameshift mutations, 8.9% in-frame indels, and 6.7% unedited alleles (Figure 1F), while the ASCT1 locus showed WT sequences only. For the ASCT1+2 KO cells, 75.8% frameshift mutations, 9.5% in-frame mutations, and 14.7% WT alleles were detected at the ASCT1 locus (Figure 1G). Similarly, at the ASCT2 locus 78.5% frameshift mutations, 11.4% in-frame indels, and 10.1% WT alleles were found. In conclusion, the genetic analysis confirmed strong enrichment of loss-of-function mutations after CRISPR-Cas9-mediated gene disruption and selection via resistance to infection through BaEVRLess- or RD114TR-pseudotyped vectors. Indel frequencies and the resistance to infection with RD114TR or BaEVRLess-pseudotyped vectors remained stable after cultivating the cell lines for over 4 months (Figures S1A and S1B).

**Figure 2:**
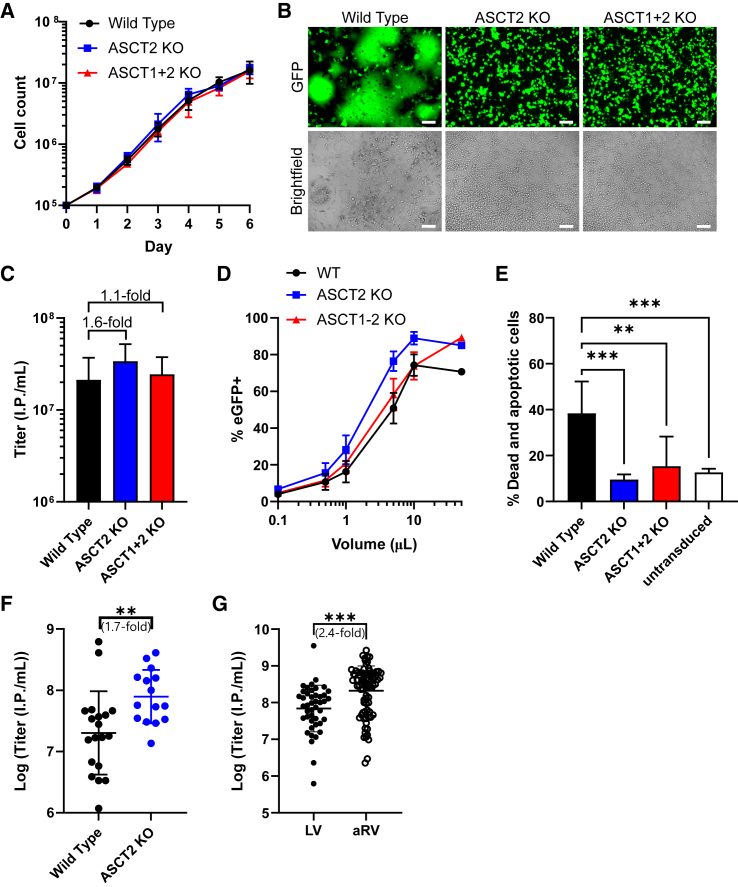
Validation of ASCT2 and ASCT1+2 KO cell lines (A) Analysis of the proliferative capacity of WT, ASCT2 KO, and ASCT1+2 KO cell lines. (B) Microscopic pictures of WT (left), ASCT2 KO (middle), and ASCT1+2 KO cell lines (right) during vector production of BaEVRLess-pseudotyped viral particles. Scale bars, 100 μm. (C) Titers of viral particles derived from WT, ASCT2 KO, and ASCT1+2 KO cells. Vectors were 100× concentrated via ultracentrifugation. (D) Titration curve of viral particles produced from WT, ASCT2 KO, or ASCT1+2 KO HEK293T cells. (E) Frequency of dead and apoptotic K562 cells 5 days after transduction with viral particles derived from WT, ASCT2 KO, or ASCT1+2 KO cell lines. (F) Titers of BaEVRLess-pseudotyped viral particles produced from WT or ASCT2 KO cell lines using a large-scale vector production and different viral vectors. Vectors were 700× concentrated via ultracentrifugation. (G) Titer comparison of lentiviral (LV) and α-retroviral (αRV) vectors pseudotyped with BaEVRLess. Statistics: mean ± SD or SEM (D), *n* = 3–9 (if not otherwise indicated by dot plot). (C and E) One-way ANOVA and (F and G) Student’s t test.

Analysis of the proliferation behavior of the cell lines revealed no differences compared with parental WT HEK293T (Figure 2A). To evaluate the consequences of the ASCT2 KO and ASCT1+2 KO on vector production, the cells were used to generate BaEVRLess-pseudotyped αRV particles. We observed syncytia formation of WT cells during vector production, while ASCT1+2 KO cells lacked cell fusions (Figure 2B). Surprisingly, the cells carrying only ASCT2 KO also lacked cell fusions despite an intact ASCT1 locus, which could serve as a second entry receptor. Consistent with this observation, ASCT2 KO cells were resistant to transduction with BaEVRLess-pseudotyped vectors (Figure S2). ASCT1+2 KO cells could be rendered amenable to infection with BaEVRLess-enveloped vectors through recombinant overexpression of ASCT1 (linked to mCherry via T2A) (Figure S3A). Using three different promoters (SFFV, PGK, and EFS) that mediate different ASCT1 expression levels, we observed ASCT1 dose-dependent susceptibility to BaEVRLess transduction in overexpressing cells (Figures S3B and S3C). To assess the impact of receptor KO on vector production, we generated BaEVRLess-pseudotyped αRV vectors produced from WT, ASCT2 KO, and ASCT1+2 KO cells (100× concentrated via ultracentrifugation). Titration on HEK293T cells revealed a 1.6-fold increase in viral titers for the ASCT2 KO cell line and a 1.1-fold titer increase for the ASCT1+2 KO cell line, indicating a moderate but consistent enhancement in viral titers (Figures 2C and 2D). Due to the elimination of syncytia formation and consequently reduced cell death and decreased accumulation of cell debris, we expected reduced cytotoxicity of concentrated viral supernatants. The cytotoxicity of the viral supernatants was assessed by transduction of K562 cells at a high multiplicity of infection (MOI) of 30, followed by measuring the amount of dead and apoptotic cells after 5 days in culture. K562 cells transduced with viral particles produced from WT, ASCT2 KO, or ASCT1+2 KO cells were >99% eGFP+ (data not shown). Compared with cells transduced with viral supernatants from WT cells, viral supernatants from ASCT2 or ASCT1+2 KO cells caused significantly less cell death and apoptosis with levels similar to untransduced K562 cells (Figure 2E). We subsequently focused on the ASCT2 KO line, and consistently obtained significantly higher titers than with unmodified HEK293T after transient transfection in larger-scale vector productions and 700× concentration via ultracentrifugation (Figure 2F). Next, we compared the relative potential of ASCT2 KO cells for the production of αRV or LV particles. αRV vector preparations consistently produced higher titers, suggesting better compatibility with the BaEVRLess glycoprotein (Figure 2G). As ASCT2 KO alone was sufficient to prevent syncytia formation, reduce cytotoxicity of viral supernatants, and generate slightly higher titers than ASCT1+2 KO cells, the ASCT2 KO cell line was chosen to generate a stable packaging cell line. In summary, the overall best performance in transient vector production using the BaEVRLess glycoprotein was observed with ASCT2 KO cells (A2) in combination with αRV vectors.

### Generation of a stable BaEVRLess αRV packaging cell line

**Figure 3:**
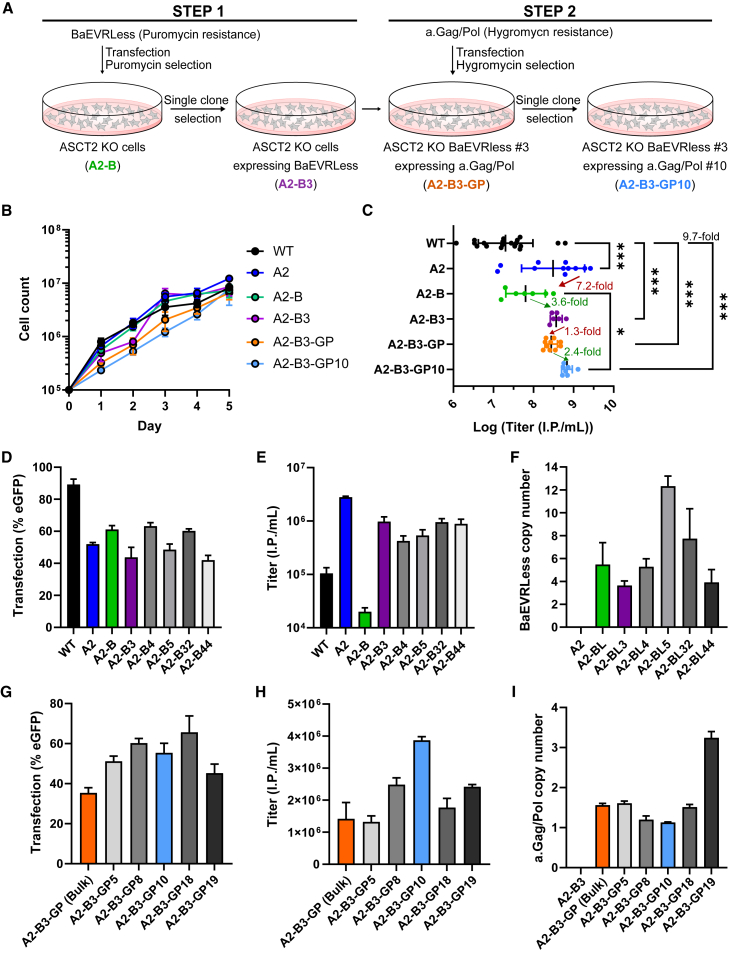
Generation of a stable BaEVRLess α-retroviral packaging cell line (A) Schematic of the procedure for stable insertion of BaEVRLess and a.Gag/Pol followed by clone selection. (B) Analysis of the proliferative capacity by assessing the cell counts of the ASCT2 KO (A2) cell line, the ASCT2 KO BaEVRLess-expressing bulk cell line (A2-B), the clonal ASCT2 KO BaEVRLess-expressing cell line (A2-B3), the ASCT2 KO BaEVRLess- and a.Gag/Pol-expressing bulk cell line (A2-B3-GP), and the clonal ASCT2 KO BaEVRLess- and a.Gag/Pol-expressing line (A2-B3-GP10) over time. (C) Titration of viral particles produced from A2, A2-B, A2-B3, A2-B3-GP, and A2-B3-GP10 cells. Vectors were 700× concentrated via ultracentrifugation. (D) After integration of the BaEVRLess cassette, 47 clones were generated and screened for a high titer-producing clone. The graph depicts the transfection rate during vector production of the top 5 clones compared with the parental lines. (E) Titers of unconcentrated viral particles produced from the top 5 selected A2-B clonal cell lines and their parental cell lines. (F) Determination of the BaEVRLess copy number of clonal A2-B lines and their parental cell lines by quantitative PCR. (G) Upon subsequent integration and selection of a.Gag/Pol-expressing A2-B3-GP cells, 20 clones were generated and screened for a high titer-producing clone. The graph depicts the transfection rate during vector production of the top 5 clones compared with the parental lines. (H) Titers of unconcentrated viral particles produced from the top 5 selected A2-B3-GP clonal cell lines and their parental bulk cell line. (I) Determination of the a.Gag/Pol copy number of the clonal A2-B3-GP lines and their parental cell line by digital droplet PCR. Statistics: all graphs represent mean ± SD, *n* = 3 (if not otherwise indicated by dot plot). (C) One-way ANOVA, (D–I) one-way ANOVA (all statistical comparisons are shown in Tables S2–S7).

For the generation of a stable BaEVRLess αRV packaging cell line, first the CMV.BaEVRLess.T2A.PuroR expression cassette was stably integrated into A2 cells using the sleeping beauty transposon system.^32^^,^^33^ The A2-B cell population (bulk cells) stably expressing the BaEVRLess glycoprotein was established via puromycin selection (Figure 3A, step 1). The proliferative capacity of bulk A2-B cells was similar to the parental A2 cell line (Figure 3B). Titration of various supernatant batches after transfection of the transfer vector pAS.SF.EGFP.PRE and a.Gag/Pol showed 7-fold lower viral titers using the bulk A2-B cell line compared with A2 cells (700× concentrated via ultracentrifugation) (Figure 3C). To overcome this reduction in titers, we performed single-cell cloning of bulk A2-B cells and screened 47 clones for a high titer-producer clone after transient transfection of the remaining vector components (data not shown). The top 5 clones were picked for a more detailed performance analysis using unconcentrated viral supernatant (A2-B clones 3, 4, 5, 32, and 44). First, we assessed the transfection rates since this parameter can strongly impact vector titers. The proportions of fluorescent cells were similar for the five clones and the A2 and A2-B parental cell lines, ranging from 40% to 60% (Figure 3D), while the transfection rate of WT HEK293T cells was about 90%. However, this observation is likely related to self-infection of WT HEK293T cells resulting in a higher frequency of eGFP+ cells, while ASCT2 KO cells are resistant to self-infection. Clone A2-B3 generated slightly higher titers among the tested clones and was used for further development (Figure 3E). Several large-scale vector productions confirmed a 3.6-fold increase in viral titers using the A2-B3 clone compared with the A2-B bulk cells, elevating titers to levels similar to transient transfection of all vector components in A2 cells (Figure 3C). Copy-number analysis revealed that clone A2-B3 contains four copies of the BaEVRLess expression cassette (Figure 3F).

In the second step, the codon-optimized a.Gag/Pol coding sequence was stably integrated into the A2-B3 cell line via plasmid transfection and hygromycin selection to generate the stable a.Gag/Pol-expressing bulk A2-B3-GP cell population (Figure 3A, step 2).^21^ The A2-B3-GP bulk population showed unchanged proliferative capacity (Figure 3B). Overall, the titers of the bulk A2-B3-GP packaging cells transfected with pAS.SF.EGFP.PRE remained slightly below the parental A2-B3 cell line (Figure 3C). Subsequently, we generated and screened clones of A2-B3-GP cells and carried out a detailed analysis of the top 5 clones for their potential to produce high viral titers using unconcentrated supernatants. All five clones demonstrated transfection rates of 40%–70%, resulting in higher viral titers for 4 out of 5 clones (Figure 3G). Clone A2-B-GP10 performed best, achieving 2.4-fold higher titers than the bulk A2-B3-GP cell line (Figure 3H). Analysis of the a.Gag/Pol copy number in this clone revealed a single integrant (Figure 3I). Multiple vector batches were produced that consistently demonstrated a significant increase in viral titers compared with the bulk A2-B3-GP cell line (Figure 3C), which remained stable over time (Figure S4). In summary, we generated a stable BaEVRLess αRV packaging cell line A2-B3-GP10 that only requires transient transfection of the transfer plasmid and achieves concentrated viral titers of 10^8^ to 10^9^ infectious particles/mL. Using entry receptor KO cells and clonal selection at each step during packaging cell line development, we were able to increase the overall titer of our final packaging cell line by 10-fold compared with the original WT HEK293T cell line.

### Absence of RCR particle formation in the stable BaEVRLess αRV packaging cell line

RCRs could potentially form through recombination between viral plasmid sequences, such as a.Gag/Pol, the transfer vector, and envelope coding sequences. To test for RCR formation in the A2-B3-GP10 packaging cell line, we transduced WT HEK293T cells with 3 × 10^8^ pAS.SF.EGFP.PRE infectious viral particles. After 2 weeks of cultivation, which allowed for the propagation and enrichment of potential RCR particles, the conditioned culture medium was transferred to fresh HEK293T cells for two consecutive rounds (Figure S5A). No eGFP+ cells were detected 3 days after transfer, as analyzed by flow cytometry. ddPCR confirmed the absence of a.Gag sequences in their genomic DNA (Figure S5B). These data demonstrate that our stable BaEVRLess αRV packaging cell line does not produce RCR within the detection limits of the assays.

### Overexpression of CD47 reduces non-specific uptake of viral particles by macrophages

**Figure 4:**
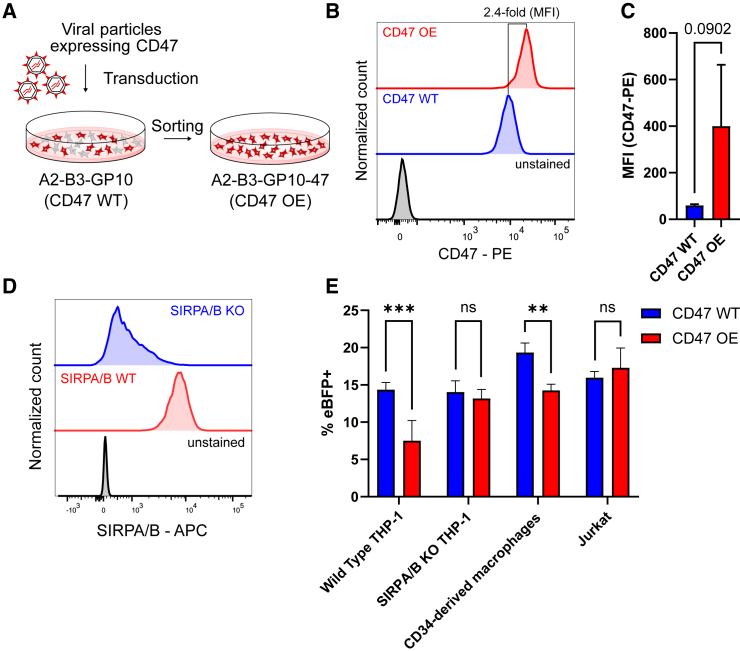
Overexpression of CD47 on the stable BaEVRLess α-retroviral packaging cell line (A) Schematic of generating a CD47-overexpressing stable packaging cell line using a lentiviral vector and cell sorting. (B) Flow cytometry histogram showing the overexpression of CD47 on sorted HEK293T cells (red) compared with endogenous expression levels (blue) and unstained cells (black). (C) Flow cytometry-based analysis of the median fluorescence intensity (MFI) of CD47 expression of α-retroviral particles generated from WT HEK293T cells expressing endogenous CD47 levels (CD47 WT) or from the CD47-overexpressing packaging cell line (CD47 OE). (D) Flow cytometric analysis of SIRPA/B expression on sorted SIRPA/B KO THP-1 cells (blue) compared with SIRPA/B WT THP-1 cells (red) and unstained THP-1 cells (black). (E) WT and SIRPA/B KO THP-1 macrophages, as well as CD34-derived primary macrophages and non-phagocytic Jurkat cells were transduced with eBFP-encoding viral particles from CD47 WT or CD47 OE packaging cell lines. The frequency of eBFP+ cells was assessed 4 days after transduction. Statistics: bar graphs represent mean ± SD. (C) Student’s t test and (E) two-way ANOVA comparing row means only.

BaEVRLess-pseudotyped viral particles are potentially useful for *in vivo* gene delivery applications due to their serum resistance and superior gene transfer into therapeutically relevant target cell populations, including lymphocytes and quiescent HSCs.^15^^,^^16^^,^^34^ Macrophages have been shown to sequester viral particles and stimulate immune responses *in vivo*, which can be partially suppressed by the incorporation of CD47 into viral particles to transmit a “don’t eat me” signal upon binding to its cognate receptor SIRPA on macrophages.^28^ To this end, we constitutively overexpressed CD47 in the A2-B3-GP10 packaging cells via transduction with the LV vector pCCL.SFFV.hCD47co.pre. Staining for CD47 and enrichment via cell sorting resulted in A2-B3-GP10-47 bulk packaging cells (Figure 4A). Endogenous CD47 expression is high in HEK293T cells, and recombinant CD47 overexpression in the A2-B3-GP10-47 packaging cells (designated CD47 OE) led to a further 2.4-fold increase in CD47 expression levels (median fluorescence intensity 9,187 vs. 22,229; Figure 4B). To confirm that CD47 was incorporated into the membrane of viral particles, we performed nano-flow analysis. Viral particles produced from CD47 OE cells (A2-B3-GP10-47 packaging cells) demonstrated a 6.7-fold increase in their median fluorescence intensity for CD47 compared with viral particles derived from the parental A2-B3-GP10 packaging cell line (Figure 4C). The ability to suppress non-specific uptake of viral particles by macrophages was evaluated on WT or SIRPA/B KO THP-1-derived macrophages, which were generated by CRISPR-Cas9-mediated KO and selection via cell sorting (Figure 4D). We observed the selective reduction of CD47 OE viral particle uptake in WT THP-1 cells (Figure 4E), while transduction rates were identical between CD47 OE and CD47 WT viral particles in SIRPA/B KO cells. These findings are consistent with the hypothesis that gene transfer into THP-1-derived macrophages is mediated both by engaging ASCT1+2 and non-specific uptake, and that the latter can be inhibited via CD47 overexpression.^28^ We also demonstrated reduced transduction using CD47 OE viral particles on human CD34-derived primary macrophages but not on a lymphoid cell line, Jurkat cells, confirming that this process is specific to macrophage-mediated non-specific uptake (Figure 4E). Taken together, these findings indicate that the increased interaction of CD47 and SIRPA between viral particles and macrophages reduces the non-specific uptake of viral particles. This approach mitigates sequestration of viral particles and transduction of non-target cells *in vivo*.

### KO of β2-microglobulin reduces the immunogenicity of viral particles

**Figure 5:**
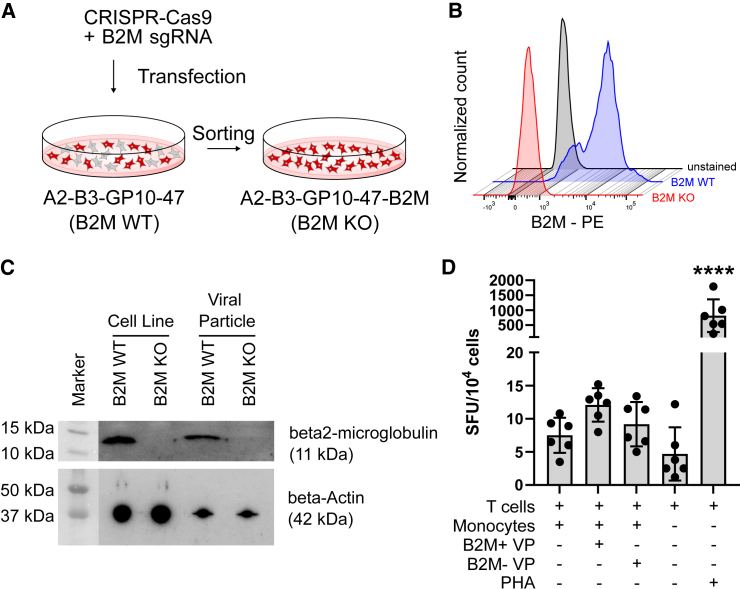
Knockout of B2M on the A2-B3-GP10-47 packaging cell line (A) Schematic of generating a B2M KO packaging cell line using CRISPR-Cas9 and cell sorting. (B) Flow cytometry histogram showing the loss of B2M expression in A2-B3-GP10-47-B2M HEK293T cells (red) compared with B2M WT cells (blue) and unstained cells (black). (C) Western blot for B2M expression in B2M WT and B2M KO packaging cells and thereof derived viral particles. β-Actin expression was used as loading control. (D) Elispot assay detecting interferon-γ-producing T cells. Monocytes were primed with viral particles derived from the B2M WT or B2M KO packaging cell line or without viral particles and subsequently co-cultured for 48 h with T cells at a 1:1 ratio. T cells only and phytohemagglutinin-stimulated T cells were used as negative and positive controls, respectively. Cells were derived from two different donors and tested at three different cell concentrations (2 × 10^5^, 1 × 10^6^, and 5 × 10^6^/mL). Spot-forming units (SFU) were normalized per 10^4^ cells for comparison. Statistics: bar graphs represent mean ± SD. One-way ANOVA revealed statistical significance only for the positive control (∗∗∗∗).

The recognition of a foreign MHC-I on the surface of viral particles or the membrane of transduced cells can potentially elicit an allo-immune response resulting in the neutralization of viral particles and transduced cells, which may be particularly relevant during *in vivo* gene transfer. As B2M is an essential component of MHC-I, loss of B2M leads to the complete absence of the MHC-I complex. To reduce the immunogenicity of viral particles, we knocked out B2M in A2-B3-GP10-47 packaging cells using transfection of an all-in-one CRISPR-Cas9 plasmid targeting B2M (Figure 5A; Barger et al.^35^), then enriched B2M-negative cells by cell sorting (Figure 5B). Complete loss of B2M on both the packaging cell line and on viral particles was confirmed by western blot (Figure 5C). To confirm that MHC-I-free viral particles are less immunogenic, we primed monocytes with viral particles derived from B2M WT (A2-B3-GP10-47 packaging cells) or B2M KO cells (A2-B3-GP10-47-B2M packaging cells), and subsequently co-cultured the primed monocytes with T cells from the same donor. The resulting T cell activation was assessed using an interferon-γ Elispot assay. After 48 h of co-cultivation, we observed a reduced number of interferon-γ spot-forming units from T cells that were co-cultured by B2M KO viral particle-primed monocytes compared with the cells that received B2M WT viral particles (Figure 5D).

### Production and testing of an MGMT-P140K-expressing vector

**Figure 6:**
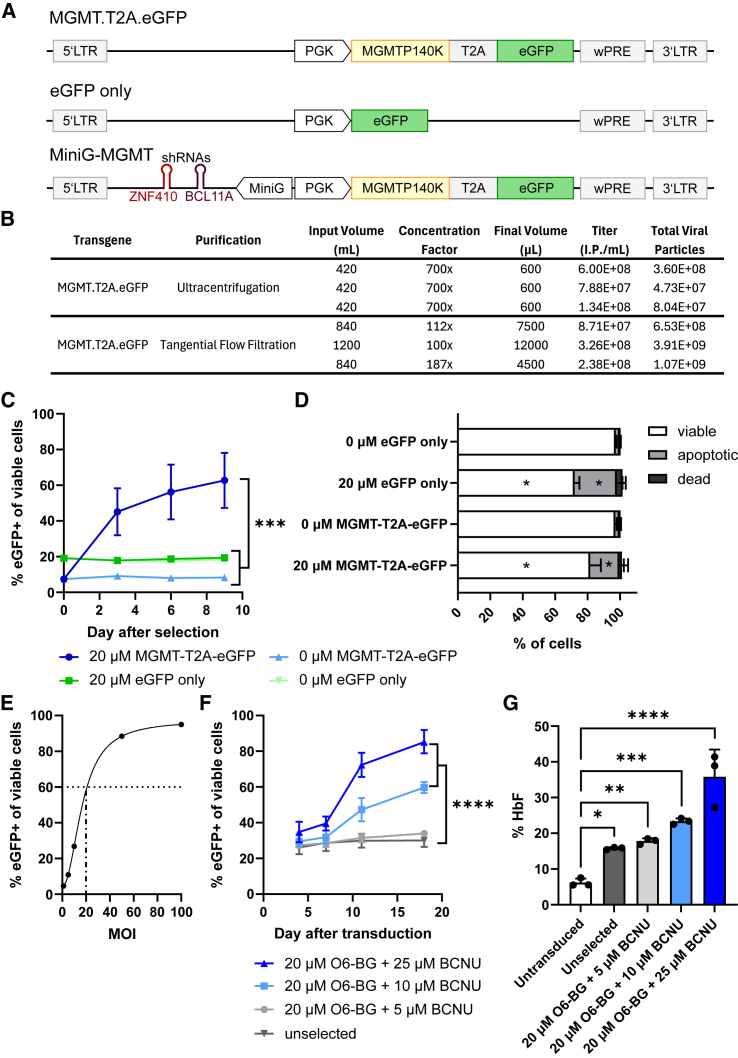
Enrichment of MGMT-P140K-expressing PLB985 cells or CD34+ HSPCs using O6BG/BCNU selection (A) Schematic of the viral vectors used to express MGMT-P140K under control of the PGK promoter. eGFP is co-expressed using a T2A peptide cleavage site (MGMT.T2A.eGFP). An eGFP-only-expressing vector was used as control (eGFP only). For CD34+ HSPCs, a therapeutic vector was used containing an erythroid-specific promoter driving the expression of two miRNA-embedded shRNAs targeting BCL11A and ZNF410 to induce fetal hemoglobin to treat sickle cell disease (MiniG-MGMT). (B) Vector production of the MGMT.T2A.eGFP vector was compared using either ultracentrifugation or tangential flow filtration to concentrate the viral supernatant. (C) PLB985 cells were transduced with the MGMT.T2A.eGFP or eGFP-only vector at a transduction rate of 10%–20%. On day 0, transduced cells were treated with a single dose of 20 μM BCNU and 10 μM O6-BG for 8 h. The enrichment of transduced cells was monitored for 9 days. Unselected cells were monitored as control. (D) The frequencies of viable, apoptotic, and dead cells were measured in the culture on day 3 of selection to confirm the induction of cell death and apoptosis in the BCNU-treated cells. (E) The MiniG-MGMT vector was tested on CD34+ HSPCs using different MOIs. The transduction rate was assessed 3 days after transduction by flow cytometry. (F) CD34+ HSPCs were transduced with the MiniG-MGMT vector at an MOI of 10 and subjected to erythroid differentiation with simultaneous BCNU selection at different doses. The enrichment of transduced cells was monitored at various time points. (G) After completion of the erythroid differentiation on day 18, cells were harvested for HPLC to assess fetal hemoglobin (HbF) induction. Statistics: *n* = 3, mean ± SD. (C, F, and G) One-way ANOVA at the respective day and (D) two-way ANOVA compared with unselected samples only.

A major limitation of *in vivo* HSC gene therapy is the relatively low transduction rate of target cells.^36^^,^^37^^,^^38^ To enrich transduced cells *in vivo,* several drug selection strategies have been developed. These include overexpression of the mutant methylguanine methyltransferase P140K (MGMT-P140K), which mediates resistance to the drug BCNU (carmustine).^36^^,^^39^^,^^40^^,^^41^ BCNU is an alkylating drug that methylates guanine nucleotides into O6-methylguanine nucleotides. These methylated nucleotides cause a DNA mismatch during DNA replication and subsequent induction of apoptosis and cell death. To test the capacity of the stable packaging cell line to produce BaEVRLess-pseudotyped, CD47-overexpressing, B2M– viral vectors suitable for *in vivo* gene transfer and selection experiments, we generated MGMT-P140K-encoding viral vectors. For simplified tracing of transduced cells, eGFP was co-expressed via a T2A element (Figure 6A), which could be replaced with any gene of interest for therapeutic applications. Instead of ultracentrifugation, which is not scalable, we tested if BaEVRLess-pseudotyped vectors could be concentrated by the scalable method of tangential flow filtration (TFF).^42^ The resulting titers (infectious particles [I.P.]/mL) were similar at approximately 10^8^ I.P./mL between UC and TFF concentrations despite up to 7-fold less volume reduction by TFF, indicating a greatly improved yield of infectious viral particles using TFF (Figure 6B). Next, we validated the MGMT vector by transducing PLB985 cells followed by chemoselection with O6-BG/BCNU. Application of a single 8 h pulse of 20 μM BCNU in combination with 10 μM O6-BG, which inhibits endogenous MGMT activity, resulted in a 6-fold enrichment of MGMT-expressing cells over 9 days compared with an eGFP-only vector (Figure 6C), which correlates with reduced dead and apoptotic cells (Figure 6D). After validation of the MGMT-P140K-expressing vector and its selection in PLB985 cells, we combined the MGMT-P140K selection vector with a therapeutic cassette designed for the erythroid-specific induction of fetal hemoglobin (HbF) via the knockdown of BCL11A and ZNF410 to treat sickle cell disease^43^ and titrated this vector (MiniG-MGMT) in CD34+ HSPCs using different MOIs (Figure 6A, bottom construct). We observed that an MOI of 20 is sufficient to transduce about 60% of CD34+ HSPCs, showing the high potency of BaEVRLess-pseudotyped viral vectors to transduce CD34+ HSPCs (Figure 6E). Finally, CD34+ HSPCs transduced at an MOI of 10 were subjected to BCNU selection at different doses and the frequency of transduced cells was measured at different time points (Figure 6F). We observed a ∼2- to 3-fold enrichment of transduced cells using 10–25 μM BCNU, respectively. Upon full erythroid differentiation after 18 days of culture, the erythroid-differentiated cells were harvested for assessment of HbF expression via HPLC. The analysis revealed a significantly higher level of HbF expression in the selected cultures, which correlates with the enrichment of gene-modified cells and reached therapeutically relevant levels (Figure 6G).

## Discussion

The central problem of syncytia formation during BaEVRLess-enveloped vector production in HEK293T cells could be addressed by the deletion of the ASCT2 entry receptor. Deletion of the second entry receptor ASCT1 appears dispensable, which is likely related to insufficient expression of ASCT1 for BaEVRLess vector uptake and syncytia formation. Supporting this hypothesis, protein expression databases indicate low ASCT1 expression in HEK293T cells (proteinatlas.org^44^), and ASCT1+2 KO cells could be rendered amenable to transduction with BaEVRLess-pseudotyped viral particles by recombinant overexpression of ASCT1 in a dose-dependent manner. Cell viability or proliferation after ASCT1/2 KO remained unaffected, which is expected due to the redundancy of neutral amino acid transporters.^45^ The resulting polyclonal ASCT2 KO cell line A2 is useful for producing BaEVRLess-pseudotyped LV and αRV vectors via transient transfection, which showed 1.7-fold improved titers, reduced variability, and toxicity of viral vector supernatants compared with WT HEK293T cells.

Similar receptor KO approaches have recently been reported for viral vectors pseudotyped with VSVg,^46^ RD114TR,^21^ BaEVRLess,^47^ or measles virus envelope (MV^48^), which overall was associated with comparable vector titer and quality improvements as observed in our study. Among these candidates, only the measles virus envelope tends to be associated with excessive fusogenicity and cell detachment during viral vector production similar to BaEVRLess. KO of the CD46 entry receptor for MV suppressed cell fusions and enhanced vector titers by approximately 2-fold, but additionally increased transduction rates of HSCs 2- to 3-fold at identical MOIs due to improved vector quality. We observed reduced cell death on cell lines when using vector produced on ASCT2 KO cells but did not evaluate the potential additional positive effect on the transduction of HSCs.

To simplify vector production, to further improve viral titers, and to show proof-of-concept for scalability, we generated a stable packaging cell line. This is relevant because the rising number of clinical trials has strained already available vector production capacities to the limit, representing a real-world barrier to the timely and cost-efficient implementation of novel therapies.^49^ Introduction of the coding sequences for BaEVRLess and a.Gag/Pol into ASCT2 KO cells, followed by the isolation of high-titer-producer clones cumulatively led to a 10-fold increase in titers, which remained remarkably stable over at least 4 months. We chose αRV instead of LV vectors because of superior titers in combination with the BaEVRLess envelope (2.4-fold) and their known compatibility with stable production systems.^21^ αRV vectors were derived from avian retroviruses with a simple genomic structure and are used in a self-inactivating long terminal repeat configuration as an added safety feature. Vector titers, compatibility with diverse envelope glycoproteins, packaging limit, and the ability to transduce quiescent cells is similar to LV vectors. A clear benefit of αRV vectors is their favorable integration pattern, which is more neutral than γ-retroviral or LV vectors,^50^^,^^51^ resulting in the lowest propensity to insertional mutagenesis.^52^^,^^53^ This makes αRV vectors the potentially safest delivery modality among retroviral vectors.

The additional overexpression of CD47 and KO of B2M in vector producer cells was well tolerated and is expected to improve the performance particularly during *in vivo* gene transfer. Both modifications have been previously explored for *in vivo* gene transfer of VSVg-pseudotyped LV vectors.^28^^,^^29^^,^^30^ Overexpression of CD47 resulted in prolonged persistence of viral particles in the circulation, reduced uptake in liver and spleen macrophage populations, reduced innate immune responses, and increased on-target transduction. For liver targeting, viral vectors derived from CD47-overexpressing producer cells was associated with 4-fold improved gene transfer rates to liver cells and a remarkable 30-fold reduction of vector genomes in Kupffer cells in NOD mice.^28^ Our own experiments and published data show a 1.5- to 3-fold reduction of uptake of CD47-overexpressing particles by macrophages *in vitro*, indicating that the effectiveness of this modification is much more pronounced *in vivo* after intravenous vector injection. Consistent with the previous report, B2M KO did not completely abolish T cell activation *in vitro* as other viral components can still trigger immune responses.^54^^,^^55^ The reduced immunogenicity could be beneficial to achieve higher on-target cell transductions and could potentially reduce the likelihood of a long-lasting memory T cell response against the viral vector, which could allow re-administration of viral vectors in an *in vivo* gene therapy setting.

The final cell line A2-B3-GP10 can easily be converted from a packaging cell line into a stable producer cell line by inserting an αRV transfer vector of choice, which eliminates the need for plasmid transfection for vector production entirely. This constitutes a major advancement for clinical studies regarding the complexity and costs of vector production. Intermediate cell lines, such as ASCT2 KO only (A2) or ASCT2 KO/BaEVRLess transgenic (A2-B3) can be used for the production of alternative viral vectors via transient transfection, such as LV vectors, or used as substrates for stably integrating the components needed for the vector system of interest.^29^^,^^56^ Further genetic modifications could be introduced to enhance viral titers, such as PKR, OAS1, TTLL12, or Drosha KO and SPT4 and SPT5 overexpression, which have been shown to be helpful, especially for the production of complex vectors.^46^^,^^57^^,^^58^^,^^59^ In conclusion, we show that the KO of ASCT2 overcomes the problems associated with the production of BaEVRLess-pseudotyped viral vectors. Further titer improvements were achieved through stable integration of vector components and clone selection, and CD47 overexpression and B2M KO are tailored to improve the performance in *in vivo* applications.

## Materials and methods

### Plasmids and viral vectors

The cloning strategies for all plasmids and viral vectors are described in the supplemental information. All sgRNA oligonucleotides, primer and probe sequences are listed in Table S1.

### KO of ASCT1, ASCT2, or B2M in HEK293T cells

To knock out ASCT1, ASCT2, or B2M, 10 μg pX458 plasmid encoding spCas9 and sgRNA were transfected into 5 × 10^5^ HEK293T cells using the polyethyleneimine (PEI) transfection method. For ASCT1 and ASCT2, KO cells were negatively selected in two repetitive cycles via the lack of transducibility with specific viral envelopes and cell sorting of eGFP– cells. ASCT2 KO cells were selected after saturating transduction (using an MOI of 10) with RD114TR-pseudotyped LV vectors encoding eGFP; ASCT1+2 KO cells were selected after transduction with BaEVRLess-pseudotyped LV vectors at saturating levels. KO at the endogenous locus was determined by PCR amplification and Sanger sequencing. The ASCT1 locus was amplified using the primers 5′-aggaacttttgactaaccagctct-3′ and 5′-ggcaggaggaaggagagaga-3′. For the ASCT2 locus, primers 5′-tatctccgggctgctctacc-3′ and 5′-tcctgaagtatggcccctgt-3′ were used. To enrich B2M KO cells, transfected cells were stained with a B2M-PE antibody and sorted for PE– cells.

### Production and titration of retroviral particles

To produce αRV particles, 1.5 × 10^7^ HEK293T cells were seeded on 15 cm plates 1 day in advance. For a three-plasmid transfection, 17.5 μg transfer plasmid, 15 μg a.Gag/Pol, and 14 μg envelope plasmid (BaEVRLess, RD114TR or VSVg) were mixed with linear PEI (Sigma-Aldrich) at a 1:5 ratio (μg plasmid/mg PEI) in 2.5 mL basal DMEM (Cytiva) medium on the day of transfection. The mixture was incubated for 20 min at room temperature and added to the HEK293T cells. After 8 h of incubation, the cells were washed with PBS, and 17.5 mL DMEM supplemented with 10% fetal bovine serum (FBS, Gemini) and 1% penicillin and streptomycin (Gibco) was added to the plate. The viral supernatant was collected 48 h after transfection, concentrated via ultracentrifugation, and resuspended in Stem Cell Growth Medium (CellGenix) with 0.5% bovine serum albumin (Invitrogen). For the stable BaEVRLess packaging cell line, 17.5 μg transfer plasmid and 15 μg a.Gag/Pol were used. For the stable BaEVRLess + a.Gag/Pol packaging cell line, 45 μg transfer plasmid was used. To produce LV particles, 25 μg transfer vector, 30 μg lenti.Gag/Pol, 15 μg Rev, and 15 μg envelope plasmid were mixed. For purification using TFF, viral supernatants were harvested twice from the packaging cells (48 and 72 h post-transfection) and purified using a 300 kDa mPES hollow fiber on the KR2i system (Repligen) by running a concentration/diafiltration/concentration cycle. All retroviral particles were titrated on 1 × 10^5^ HEK293T cells, for which 5 × 10^4^ HEK293T cells/well were seeded 1 day in advance in 48-well plates. On the day of titration, a serial dilution of the concentrated viral supernatant was applied to the HEK293T cells. The transduction rate was measured 3 days post-transduction by flow cytometry.

### Proliferation assay

The proliferation of different cell lines was assessed by quantification of absolute cell counts. On day 0, 1 × 10^5^ cells were seeded in triplicate in 6-well plates. Each day, the viable cell count of one well was measured using trypan blue staining and an automated cell counter (Countess III).

### Cytotoxicity assay

K562 cells were transduced at an MOI of 100 and cultured for 5 days to analyze the cytotoxicity of concentrated viral supernatants. The cells were stained with Annexin V-APC for 15 min in 1× Annexin V staining buffer at room temperature. DAPI was added to the cells, and the transduction rate and frequency of dead and apoptotic cells were assessed via flow cytometry on a Fortessa cytometer (BD).

### Stable integration of BaEVRLess and a.Gag/Pol

The sleeping beauty system was used to stably integrate the BaEVRLess expression cassette into the ASCT2 KO cell line (A2). One day before transfection, 5 × 10^5^ A2 cells were seeded in a 6-well plate. The cells were transfected with 5 μg SB100x transposase and 5 μg pT4.CMV.BaEVRLess.T2A.PuroR.bGHpA (B) using the PEI transfection method.^32^^,^^33^ Three days after transfection, the generated A2-B cells were selected with 5 μg/mL puromycin for 1 week. In a subsequent step, the pSK.CAG.a.Gag/Pol(co).IRES.HygroR.pA plasmid (GP) was delivered by transfection of 10 μg expression plasmid using the PEI transfection method. Three days after transfection, the generated A2-B-GP cells were selected with 100 μg/mL hygromycin for 1 week.

### Screening for a high-titer clone

A2-B or A2-B3-GP cells were seeded at a concentration of 0.5 cells/well into a total of five 96-well flat-bottom plates to identify a high titer clone. The single-cell clones were cultured for 2 weeks and further expanded for viral vector production. During the screening process, vector production was performed in a 6-well format using 1 × 10^6^ cells per well in triplicate. One day after seeding, cells were transfected with 1 μg pAS.SF.EGFP.PRE ± 1 μg GP using the PEI method and incubated overnight. The medium was changed to 1 mL supplemented medium the next day. Viral supernatants were harvested 32 h after the medium change and filtered through a 0.22-μm PVDF filter. Unconcentrated viral supernatants were titrated on 1 × 10^5^ 293T cells.

### Determination of BaEVRLess and a.Gag/Pol copy numbers

Genomic DNA was isolated using the DNeasy Blood and Tissue Kit (QIAGEN). BaEVRLess copies were amplified using the primers 5′-agggcagtctatttgctgga-3′ and 5′-ggccaaagggtgatactgaa-3′ and the SYBR Green PCR Master Mix (Applied Biosystems). Copies were quantified by quantitative real-time PCR using the QuantStudio 3 PCR cycler (Applied Biosystems) and normalized to human albumin copies, which were amplified using the primers 5′-gctgtcatctcttgtgggctgt-3′ and 5′-actcatgggagctgctggttc-3′. The a.Gag/Pol copy number in the packaging cell lines and for testing of RCR vectors was determined by digital droplet PCR (ddPCR, BioRad) using the ddPCR Supermix for Probes (no dUTP) (Bio-Rad). The a.Gag/Pol was amplified using the primers 5′-ccagcaagaaagaaatcggc-3′ and 5′-ggtcacctgttcttctctgg-3′ and the probe 5′-FAM-gccgccctgagccagagggc-BHQ-3′. The a.Gag/Pol copies were normalized to human albumin using the primers described above and the probe 5-HEX-cctgtcatgcccacacaaatctctcc-BHQ-3′.

### Overexpression of human CD47

The stable packaging cell line was transduced with the pCCL.SFFV.hCD47co.pre vector to overexpress the “don’t eat me” signal CD47. After transduction, cells were stained with anti-human CD47-PE (BioLegend) and sorted for high-expressing cells on an Aria cell sorter (BD). Viral particles were produced from the CD47-overexpressing cell line. To analyze the increased presence of CD47 molecules on the surface of the viral particles, 10^7^ viral particles were stained with 10 μL anti-human CD47-PE antibody for 30 min and analyzed on a CytoFlex cytometer (Beckman Coulter).

### Macrophage transduction assay

To analyze the effect of CD47 overexpression on viral particles on the transduction of macrophages, viral particles produced from HEK293T cells with endogenous CD47 expression levels (CD47 WT) and CD47-overexpressing cells (CD47 OE) were used to transduce the macrophage cell line THP-1. As a genetic control, SIRPA KO THP-1 cells were generated by transducing WT cells with an inducible all-in-one CRISPR-Cas9 LV vector. Cas9 expression was induced 3 days after transduction by adding 0.1 μg/mL doxycycline (Sigma-Aldrich). KO cells were stained using an anti-human SIRPA/B-APC antibody (BioLegend) and sorted for SIRPA/B– cells using the FACS Melody sorter (BD). THP-1 cells were terminally differentiated using 100 μg/mL PMA (Sigma-Aldrich) for 24 h. After differentiation, 10^5^ WT and SIRPA KO THP-1 cells were seeded into a 48-well plate and transduced at an MOI of 1 with a BaEVRLess-pseudotyped eBFP-expressing αRV vector (aRV.SBW) produced on either CD47 WT or CD47 OE HEK293T cells. The frequency of eBFP+ cells was determined 4 days after transduction. In addition to THP-1 cells, M-CSF (Peprotech)-differentiated CD34-derived primary macrophages were tested in the macrophage transduction assay as well as Jurkat cells as a non-phagocytic cell line.

### Western blot

To confirm loss of ASCT2 or B2M expression on the packaging cell line or on the membrane of viral particles, the respective cell lines or viral particles produced from these cell lines were lysed for 15 min on ice in complete RIPA buffer (Millipore) and separated on a 4%–20% Novex Tris-Glycine Mini Protein Gel (Invitrogen). The samples were blotted onto a methanol-activated PVDF membrane (Millipore) using the XCell II Blot Module (Invitrogen). After blocking the membrane in 5% milk in TBST buffer, ASCT2 or B2M were detected using a monoclonal rabbit-anti-human ASCT2 antibody (CST, no. 8057, 1:1,000) or a monoclonal rabbit-anti-human B2M antibody (Abcam, no. 75853, 1:4,000) as primary antibody in 5% milk in TBST and an HRP-conjugated goat anti-rabbit secondary antibody (CST, no. 7074P2) 1:10,000 in 5% milk in TBST. The HRP signal was detected using the SuperSignal West Pico PLUS Chemiluminescent Substrate (Thermo Fisher) and the ChemiDoc (Bio-Rad). As loading control, the same membrane was stained with β-actin using the primary monoclonal rabbit anti-human β-actin antibody (AB clonal, AC026, 1:5,000) or a direct conjugated anti-GAPDH-HRP (Thermo Fisher, no. MA5-15738-HRP, 1:10,000) in 5% milk in TBST.

### Elispot assay

Human peripheral blood was kindly provided from the Blood Donor Center at Boston Children’s Hospital. Monocytes and T cells were isolated using the Classical Monocyte Isolation Kit (Miltenyi) and the Pan T cell Isolation Kit (Miltenyi), respectively. On day 0, 2.5 × 10^5^ monocytes were incubated for 16 h with viral particles, derived from either the B2M WT or KO packaging cell lines, at an MOI of 100 or without viral particles in IMDM (Cytiva) supplemented with 10% FBS, 100 IU/mL penicillin and streptomycin. T cells were cultured in the following T cell medium: IMDM supplemented with 10% FBS, 100 IU/mL penicillin and streptomycin, 2.5 mM L-glutamine, 1× non-essential amino acids (Thermo Fisher Scientific), 50 μM β-mercaptoethanol (Thermo Fisher Scientific), 20 U/mL human IL-2 (BioLegend), 5 ng/μL human IL-7 (BioLegend), and 5 ng/μL human IL-15 (BioLegend). On day 1, monocytes and T cells were washed three times with PBS. One hundred microliters of monocytes and 100 μL T cells, both at concentrations of 2 × 10^5^, 1 × 10^6^, and 5 × 10^6^/mL in T cell medium without cytokines, were seeded in a pre-coated IFN-γ ELISPOT plate (BD). After 48 h of incubation, the plate was washed and developed according to the manufacturer’s instructions. Spot development was monitored for up to 1 h before stopping the reaction with deionized water. The plate was read at the ELISPOT reader (via ZellNet Consulting) after at least 24 h of air-drying protected from direct light exposure. T cells stimulated with mitogen phytohemagglutinin (2 μg/mL; Roche Diagnostics) and T cell only were used as positive and negative controls, respectively.

### O6BG/BCNU selection of MGMT-expressing cells

PLB985 cells were transduced at an MOI of 0.2 with the aRV.PMEW or pAS.SF.EGFP.PRE vector to achieve a transduction rate of 10%–20% eGFP+ cells. Transduced cells were selected using continuous administration of 10 μM O6-BG (Sigma) and 20 μM BCNU (Sigma). The enrichment of MGMT-expressing cells was monitored by assessing the frequency of eGFP-expressing cells via flow cytometry on day 3, 6, and 9 after start of selection. On day 3, the frequency of dead and apoptotic cells was measured using Annexin V/DAPI staining as described above. For CD34+ HSPC transduction and selection, we used the therapeutic vector MiniG-MGMT and first tested this vector on CD34+ HSPCs using MOIs of 1, 5, 10, 50, and 100. Next, CD34+ HSPCs transduced with the MOI of 10 were subjected to *in vitro* erythroid differentiation as described elsewhere^43^ with simultaneous BCNU selection at doses of 20 μM O6-BG plus 5, 10, or 20 μM BCNU. At the end of erythroid differentiation at day 18, 10^6^ erythroid cells were lysed for 15 min using Hemolysate reagent (Helena Laboratories). After centrifugation at 16,000 × g for 5 min, the lysate was diluted 1:8 in D-10 Buffer (Bio-Rad) and analyzed on the D-10 Hemoglobin Analyzer (Bio-Rad) for the quantification of hemoglobin variants.

### Statistical analysis

All graphs represent the mean ± SD. After confirming parametric data distribution, Student’s t test for comparing two groups or one-way ANOVA for comparing multiple groups was used. Statistical significance is indicated by an asterisk. All statistics were done with GraphPad Prism 9.

## Data and code availability

The data that support the findings of this study, plasmids, and cell lines are available on request from the corresponding author (C.B.).

## Acknowledgments

We thank Kayla E. Wright for technical assistance. This research was funded by the 10.13039/100000865Bill and Melinda Gates Foundation (INV-021791 and INV-050202, to C.B. and D.A.W.), the 10.13039/501100001659Deutsche Forschungsgemeinschaft (10.13039/501100001659German Research Foundation, 451828430, to D.K.), R01HL170629 (to P.G.), and R01HL172489 (to C.B.).

## Author contributions

D.K. and C.B. designed the experiments and wrote the manuscript. D.K. executed and analyzed the experiments. L.S. and B.L. helped design and execute portions of the experiments. E.V., D.A.W., P.G., and A.S. advised the experiments.

## Declaration of interests

The authors declare no competing interests.

## Supplemental information

Document S1. Figures S1–S5 and Tables S1–S7

Document S2. Article plus supplemental information
