# Supplementary material for: Engineered packaging cell line for the enhanced production of baboon-enveloped retroviral vectors
Source: Mol Ther Nucleic Acids. 2024 Nov 13;35(4):102389. doi: 10.1016/j.omtn.2024.102389 (PMC11638596; doi:10.1016/j.omtn.2024.102389)
Supplement: Document S1. Figures S1–S5 and Tables S1–S7 [file mmc1.pdf]

OMTN, Volume *35*

Supplemental information

Engineered packaging cell line for the enhanced production of baboon-enveloped retroviral vectors

Denise Klatt, Lucia Sereni, Boya Liu, Pietro Genovese, Axel Schambach, Els Verhoeyen, David A. Williams, and Christian Brendel

Supplemental materials and methods

Plasmids and viral vectors

To knock out ASCT1 and ASCT2, the single guide RNAs (sgRNAs) targeting ASCT1 and ASCT2 were cloned into the pSpCas9(BB)-2A-GFP plasmid (pX458), which was a gift from Feng Zhang (Addgene plasmid # 48138; http://n2t.net/addgene:48138; RRID:Addgene_48138) [57]. For ASCT1, the oligodeoxynucleotides 5'-caccgctgatcctgcagatccccat-3' and 5'-aaacatggggatctgcaggatcagc-3' were phosphorylated for 30 minutes at 37°C using T4 polynucleotide kinase (New England Biolabs (NEB)) and subsequently annealed at 95°C for five minutes and ramped down to 22°C at a rate of -0.1°C/s. The phosphorylated and annealed oligodeoxynucleotides were ligated into the BsmBI (NEB) digested pX458 backbone. For ASCT2, the oligodeoxynucleotides 5'-caccgctgatcaggtacgcccctgt-3' and 5'-aaacacaggggcgtacctgatcagc-3' were used accordingly.

For the selection of ASCT1 and/or ASCT2 KO cells, a third-generation LV vector expressing eGFP driven by the spleen focus-forming virus (SFFV) promoter was used (pCCL.SEW). For vector production on the packaging cell line, an αRV SIN vector expressing eGFP under the control of the SFFV promoter was used (pAS.SF.EGFP.PRE) [49,58].

Overexpression of ASCT1 was achieved using a LV vector that encodes ASCT1 and the mCherry fluorescent protein separated by a T2A peptide cleavage site to track transduced cells. To induce different ASTC1 expression levels, three different promoters were exploited, namely, the SFFV promoter (supraphysiological expression levels), the phosphoglycerate kinase PGK promoter, and the short elongation factors 1α (EFS) promoter (both physiological expression levels). The ASCT1 coding sequence was obtained from pDONR221_SLC1A4, which was a gift from RESOLUTE Consortium & Giulio Superti-Furga (Addgene plasmid # 131962; http://n2t.net/addgene:131962; RRID: Addgene_131962).

To stably integrate BaEVRLess into the packaging cell, the BaEVRLess sequence including a CMV promoter was transferred from the envelope packaging plasmid [8] into the transposon plasmid pT4/HB, which was a gift from Wolfgang Uckert (Addgene plasmid # 108352; http://n2t.net/addgene:108352; RRID: Addgene_108352) [31]. A puromycin selection cassette was included downstream of BaEVRLess separated by a T2A peptide cleavage site. The transposon was named pT4.CMV.BaEVRLess.T2A.PuroR.bGHpA. The SB100x transposase plasmid pCMV(CAT)T7-SB100 was a gift from Zsuzsanna Izsvak (Addgene plasmid # 34879; http://n2t.net/addgene:34879; RRID: Addgene_34879) [32].

For the stable integration of αRV a.Gag/Pol, the plasmid pSK.CAG.aGag/Pol(co).IRES.HygroR.pA [20] was transfected into the ASCT2 KO + BaEVRLess #3 cell line using the PEI transfection method.

To overexpress CD47, the human codon-optimized CD47 was synthesized by Genewiz (Azenta Life Sciences) and cloned into a third-generation LV vector behind the SFFV promoter generating the pCCL.SFFV.hCD47co.pre vector.

To knock out SIRPA on THP-1 cells, a sgRNA targeting SIRPA (and SIRPB due to their high sequence homology) was cloned into the TLCV2 plasmid, which is an inducible all-in-one CRISPR-Cas9 vector (TLCV2 was a gift from Adam Karpf (Addgene plasmid # 87360; http://n2t.net/addgene:87360; RRID: Addgene_87360)) [34]. The phosphorylated and annealed oligonucleotides 5'-caccgtccctgtggggcccatccag-3' and 5'-aaacctggatgggccccacagggac-3' were cloned into the BsmBI digested TLCV2 backbone as described above.

To transduce THP-1 macrophages, an αRV vector expressing eBFP under the control of the SFFV promoter was used (aRV.SBW).

To knock out B2M on HEK293T cells, a sgRNA was cloned into pX458 as described above using the oligonucleotides 5'-caccgagtagcgcgagcacagcta-3' and 5'-aaactagctgtgctcgcgctactc-3'.

For O6BG/BCNU selection the MGMT-P140K cassette was cloned downstream of the PGK promoter followed by T2A.eGFP to track transduced cells generating the aRV.PMEW vector. For CD34+ HSPC transduction, a lentiviral vector containing the PGK.MGMT-P140K.T2A.eGFP expression cassette was used and an erythroid-specific minimal beta-globin promoter (MiniG) expressing two miRNA-embedded shRNAs targeting BCL11A and ZNF410 was cloned upstream of the PGK promoter in reverse orientation generating the vector MiniG-MGMT.

Testing for replication-competent retroviral particles (RCR)

RCR testing was performed in triplicates by transducing 10^7 HEK293T cells with 10^8 pAS.SF.EGFP.PRE viral particles produced from the stable A2-B3-GP10 packaging cell line. The transduced cells were cultured for two weeks to allow the enrichment of potential replication-competent viral particles. In the first transfer, conditioned medium was harvested from the initial culture, filtered through a 0.22 µm filter and added to 5×10^5 HEK293T cells. After overnight incubation, the medium was changed to fresh supplemented medium, and the cells were cultured for three days. The conditioned medium was filtered and transferred to 5×10^5 HEK293T cells for the second transfer. Three days after the first and second transfer, the HEK293T cells were assessed by flow cytometry for eGFP-positive cells and genomic DNA was extracted to test for a.Gag/Pol copies by ddPCR.

Table S1: Sequences for sgRNAs oligos, primers and probes

Name	Sequence (5'-3')
**sgRNA oligos**	
**ASCT1_sgRNA_fw**	caccgctgatcctgcagatccccat
**ASCT1_sgRNA_rev**	aaacatggggatctgcaggatcagc
**ASCT2_sgRNA_fw**	caccgctgatcaggtacgcccctgt
**ASCT2_sgRNA_rev**	aaacacaggggcgtacctgatcagc
**SIRPA_sgRNA_fw**	caccgtccctgtggggcccatccag
**SIRPA_sgRNA_rev**	aaacctggatgggccccacagggac
**B2M_sgRNA_fw**	caccgagtagcgcgagcacagcta
**B2M_sgRNA_rev**	aaactagctgtgctcgcgctactc
**PCR**	
**ASCT1_fw**	aggaacttttgactaaccagctct
**ASCT1_rev**	ggcaggaggaaggagagaga
**ASCT2_fw**	tatctccgggctgctctacc
**ASCT2_rev**	tcctgaagtatggcccctgt
**BaEVRLess_fw**	agggcagtctatttgctgga
**BaEVRLess_rev**	ggccaaagggtgatactgaa
**Albumin_fw**	gctgtcatctcttgtgggctgt
**Albumin_rev**	actcatgggagctgctggttc
**Albumin_probe**	cctgtcatgcccacacaaatctctcc
**a.Gag/Pol_fw**	ccagcaagaaagaaatcggc
**a.Gag/Pol_rev**	ggtcacctgttcttctctgg
**a.Gag/Pol_probe**	gccgccctgagccagagggc

Table S2: Statistical analysis of Figure 3D.

Number of families	1					
Number of comparisons per family	28					
Alpha	0.05					
Tukey's multiple comparisons test	Mean Diff.	95.00% CI of diff.	Below threshold?	Summary	Adjusted P Value	
WT vs. A2	37.20	27.98 to 46.42	Yes	****	<0.0001	A-B
WT vs. A2-BL	28.03	18.81 to 37.25	Yes	****	<0.0001	A-C
WT vs. A2-BL3	45.47	36.25 to 54.69	Yes	****	<0.0001	A-D
WT vs. A2-BL4	25.97	16.75 to 35.19	Yes	****	<0.0001	A-E
WT vs. A2-BL5	40.63	31.41 to 49.85	Yes	****	<0.0001	A-F
WT vs. A2-BL32	29.00	19.78 to 38.22	Yes	****	<0.0001	A-G
WT vs. A2-BL44	47.23	38.01 to 56.45	Yes	****	<0.0001	A-H
A2 vs. A2-BL	-9.167	-18.39 to 0.05419	No	ns	0.0519	B-C
A2 vs. A2-BL3	8.267	-0.9542 to 17.49	No	ns	0.0963	B-D
A2 vs. A2-BL4	-11.23	-20.45 to -2.012	Yes	*	0.0117	B-E
A2 vs. A2-BL5	3.433	-5.788 to 12.65	No	ns	0.8902	B-F
A2 vs. A2-BL32	-8.200	-17.42 to 1.021	No	ns	0.1007	B-G
A2 vs. A2-BL44	10.03	0.8125 to 19.25	Yes	*	0.0280	B-H
A2-BL vs. A2-BL3	17.43	8.212 to 26.65	Yes	***	0.0001	C-D
A2-BL vs. A2-BL4	-2.067	-11.29 to 7.154	No	ns	0.9922	C-E
A2-BL vs. A2-BL5	12.60	3.379 to 21.82	Yes	**	0.0043	C-F
A2-BL vs. A2-BL32	0.9667	-8.254 to 10.19	No	ns	>0.9999	C-G
A2-BL vs. A2-BL44	19.20	9.979 to 28.42	Yes	****	<0.0001	C-H
A2-BL3 vs. A2-BL4	-19.50	-28.72 to -10.28	Yes	****	<0.0001	D-E
A2-BL3 vs. A2-BL5	-4.833	-14.05 to 4.388	No	ns	0.6200	D-F
A2-BL3 vs. A2-BL32	-16.47	-25.69 to -7.246	Yes	***	0.0003	D-G
A2-BL3 vs. A2-BL44	1.767	-7.454 to 10.99	No	ns	0.9970	D-H
A2-BL4 vs. A2-BL5	14.67	5.446 to 23.89	Yes	***	0.0010	E-F
A2-BL4 vs. A2-BL32	3.033	-6.188 to 12.25	No	ns	0.9380	E-G
A2-BL4 vs. A2-BL44	21.27	12.05 to 30.49	Yes	****	<0.0001	E-H
A2-BL5 vs. A2-BL32	-11.63	-20.85 to -2.412	Yes	**	0.0087	F-G
A2-BL5 vs. A2-BL44	6.600	-2.621 to 15.82	No	ns	0.2711	F-H
A2-BL32 vs. A2-BL44	18.23	9.012 to 27.45	Yes	****	<0.0001	G-H

Table S3: Statistical analysis of Figure 3E.

Number of families	1					
Number of comparisons per family	28					
Alpha	0.05					
Tukey's multiple comparisons test	Mean Diff.	95.00% CI of diff.	Below threshold?	Summary	Adjusted P Value	
WT vs. A2	-2691967	-3087705 to -2296228	Yes	****	<0.0001	A-B
WT vs. A2-BL	84700	-311039 to 480439	No	ns	0.9940	A-C
WT vs. A2-BL3	-869300	-1265039 to -473561	Yes	****	<0.0001	A-D
WT vs. A2-BL4	-317967	-713705 to 77772	No	ns	0.1678	A-E
WT vs. A2-BL5	-431633	-827372 to -35895	Yes	*	0.0275	A-F
WT vs. A2-BL32	-847967	-1243705 to -452228	Yes	****	<0.0001	A-G
WT vs. A2-BL44	-778967	-1174705 to -383228	Yes	****	<0.0001	A-H
A2 vs. A2-BL	2776667	2380928 to 3172405	Yes	****	<0.0001	B-C
A2 vs. A2-BL3	1822667	1426928 to 2218405	Yes	****	<0.0001	B-D
A2 vs. A2-BL4	2374000	1978261 to 2769739	Yes	****	<0.0001	B-E
A2 vs. A2-BL5	2260333	1864595 to 2656072	Yes	****	<0.0001	B-F
A2 vs. A2-BL32	1844000	1448261 to 2239739	Yes	****	<0.0001	B-G
A2 vs. A2-BL44	1913000	1517261 to 2308739	Yes	****	<0.0001	B-H
A2-BL vs. A2-BL3	-954000	-1349739 to -558261	Yes	****	<0.0001	C-D
A2-BL vs. A2-BL4	-402667	-798405 to -6928	Yes	*	0.0446	C-E
A2-BL vs. A2-BL5	-516333	-912072 to -120595	Yes	**	0.0065	C-F
A2-BL vs. A2-BL32	-932667	-1328405 to -536928	Yes	****	<0.0001	C-G
A2-BL vs. A2-BL44	-863667	-1259405 to -467928	Yes	****	<0.0001	C-H
A2-BL3 vs. A2-BL4	551333	155595 to 947072	Yes	**	0.0036	D-E
A2-BL3 vs. A2-BL5	437667	41928 to 833405	Yes	*	0.0249	D-F
A2-BL3 vs. A2-BL32	21333	-374405 to 417072	No	ns	>0.9999	D-G
A2-BL3 vs. A2-BL44	90333	-305405 to 486072	No	ns	0.9913	D-H
A2-BL4 vs. A2-BL5	-113667	-509405 to 282072	No	ns	0.9687	E-F
A2-BL4 vs. A2-BL32	-530000	-925739 to -134261	Yes	**	0.0052	E-G
A2-BL4 vs. A2-BL44	-461000	-856739 to -65261	Yes	*	0.0168	E-H
A2-BL5 vs. A2-BL32	-416333	-812072 to -20595	Yes	*	0.0356	F-G
A2-BL5 vs. A2-BL44	-347333	-743072 to 48405	No	ns	0.1081	F-H
A2-BL32 vs. A2-BL44	69000	-326739 to 464739	No	ns	0.9983	G-H

Table S4: Statistical analysis of Figure 3F.

Number of families	1					
Number of comparisons per family	21					
Alpha	0.05					
Tukey's multiple comparisons test	Mean Diff.	95.00% CI of diff.	Below threshold?	Summary	Adjusted P Value	
A2 vs. A2-BL	-5.475	-9.855 to -1.095	Yes	*	0.0117	A-B
A2 vs. A2-BL3	-3.645	-8.026 to 0.7347	No	ns	0.1292	A-C
A2 vs. A2-BL4	-5.278	-9.196 to -1.361	Yes	**	0.0067	A-D
A2 vs. A2-BL5	-12.33	-16.24 to -8.410	Yes	****	<0.0001	A-E
A2 vs. A2-BL32	-7.736	-11.65 to -3.818	Yes	***	0.0002	A-F
A2 vs. A2-BL44	-3.919	-7.837 to -0.001567	Yes	*	0.0499	A-G
A2-BL vs. A2-BL3	1.830	-2.968 to 6.628	No	ns	0.8239	B-C
A2-BL vs. A2-BL4	0.1970	-4.183 to 4.577	No	ns	>0.9999	B-D
A2-BL vs. A2-BL5	-6.852	-11.23 to -2.472	Yes	**	0.0020	B-E
A2-BL vs. A2-BL32	-2.261	-6.641 to 2.119	No	ns	0.5675	B-F
A2-BL vs. A2-BL44	1.556	-2.824 to 5.936	No	ns	0.8641	B-G
A2-BL3 vs. A2-BL4	-1.633	-6.013 to 2.747	No	ns	0.8376	C-D
A2-BL3 vs. A2-BL5	-8.682	-13.06 to -4.302	Yes	***	0.0002	C-E
A2-BL3 vs. A2-BL32	-4.091	-8.471 to 0.2895	No	ns	0.0731	C-F
A2-BL3 vs. A2-BL44	-0.2738	-4.654 to 4.106	No	ns	>0.9999	C-G
A2-BL4 vs. A2-BL5	-7.049	-10.97 to -3.131	Yes	***	0.0006	D-E
A2-BL4 vs. A2-BL32	-2.458	-6.376 to 1.460	No	ns	0.3633	D-F
A2-BL4 vs. A2-BL44	1.359	-2.559 to 5.277	No	ns	0.8759	D-G
A2-BL5 vs. A2-BL32	4.591	0.6735 to 8.509	Yes	*	0.0184	E-F
A2-BL5 vs. A2-BL44	8.408	4.490 to 12.33	Yes	***	0.0001	E-G
A2-BL32 vs. A2-BL44	3.817	-0.1009 to 7.734	No	ns	0.0580	F-G

Table S5: Statistical analysis of Figure 3D.

Number of families	1					
Number of comparisons per family	15					
Alpha	0.05					
Tukey's multiple comparisons test	Mean Diff.	95.00% CI of diff.	Below threshold?	Summary	Adjusted P Value	
A2-BL3-GP (Bulk) vs. A2-BL3-GP5	-15.77	-28.56 to -2.976	Yes	*	0.0134	A-B
A2-BL3-GP (Bulk) vs. A2-BL3-GP8	-24.83	-37.62 to -12.04	Yes	***	0.0003	A-C
A2-BL3-GP (Bulk) vs. A2-BL3-GP10	-19.97	-32.76 to -7.176	Yes	**	0.0022	A-D
A2-BL3-GP (Bulk) vs. A2-BL3-GP18	-30.23	-43.02 to -17.44	Yes	****	<0.0001	A-E
A2-BL3-GP (Bulk) vs. A2-BL3-GP19	-9.867	-22.66 to 2.924	No	ns	0.1730	A-F
A2-BL3-GP5 vs. A2-BL3-GP8	-9.067	-21.86 to 3.724	No	ns	0.2364	B-C
A2-BL3-GP5 vs. A2-BL3-GP10	-4.200	-16.99 to 8.591	No	ns	0.8711	B-D
A2-BL3-GP5 vs. A2-BL3-GP18	-14.47	-27.26 to -1.676	Yes	*	0.0238	B-E
A2-BL3-GP5 vs. A2-BL3-GP19	5.900	-6.891 to 18.69	No	ns	0.6425	B-F
A2-BL3-GP8 vs. A2-BL3-GP10	4.867	-7.924 to 17.66	No	ns	0.7911	C-D
A2-BL3-GP8 vs. A2-BL3-GP18	-5.400	-18.19 to 7.391	No	ns	0.7168	C-E
A2-BL3-GP8 vs. A2-BL3-GP19	14.97	2.176 to 27.76	Yes	*	0.0191	C-F
A2-BL3-GP10 vs. A2-BL3-GP18	-10.27	-23.06 to 2.524	No	ns	0.1471	D-E
A2-BL3-GP10 vs. A2-BL3-GP19	10.10	-2.691 to 22.89	No	ns	0.1574	D-F
A2-BL3-GP18 vs. A2-BL3-GP19	20.37	7.576 to 33.16	Yes	**	0.0019	E-F

Table S6: Statistical analysis of Figure 3H.

Number of families	1					
Number of comparisons per family	15					
Alpha	0.05					
Tukey's multiple comparisons test	Mean Diff.	95.00% CI of diff.	Below threshold?	Summary	Adjusted P Value	
A2-BL3-GP (Bulk) vs. A2-BL3-GP5	97333	-644458 to 839125	No	ns	0.9973	A-B
A2-BL3-GP (Bulk) vs. A2-BL3-GP8	-1066000	-1807791 to -324209	Yes	**	0.0043	A-C
A2-BL3-GP (Bulk) vs. A2-BL3-GP10	-2449333	-3191125 to -1707542	Yes	****	<0.0001	A-D
A2-BL3-GP (Bulk) vs. A2-BL3-GP18	-346000	-1087791 to 395791	No	ns	0.6326	A-E
A2-BL3-GP (Bulk) vs. A2-BL3-GP19	-999333	-1741125 to -257542	Yes	**	0.0070	A-F
A2-BL3-GP5 vs. A2-BL3-GP8	-1163333	-1905125 to -421542	Yes	**	0.0021	B-C
A2-BL3-GP5 vs. A2-BL3-GP10	-2546667	-3288458 to -1804875	Yes	****	<0.0001	B-D
A2-BL3-GP5 vs. A2-BL3-GP18	-443333	-1185125 to 298458	No	ns	0.3918	B-E
A2-BL3-GP5 vs. A2-BL3-GP19	-1096667	-1838458 to -354875	Yes	**	0.0034	B-F
A2-BL3-GP8 vs. A2-BL3-GP10	-1383333	-2125125 to -641542	Yes	***	0.0005	C-D
A2-BL3-GP8 vs. A2-BL3-GP18	720000	-21791 to 1461791	No	ns	0.0590	C-E
A2-BL3-GP8 vs. A2-BL3-GP19	66667	-675125 to 808458	No	ns	0.9996	C-F
A2-BL3-GP10 vs. A2-BL3-GP18	2103333	1361542 to 2845125	Yes	****	<0.0001	D-E
A2-BL3-GP10 vs. A2-BL3-GP19	1450000	708209 to 2191791	Yes	***	0.0003	D-F
A2-BL3-GP18 vs. A2-BL3-GP19	-653333	-1395125 to 88458	No	ns	0.0969	E-F

Table S7: Statistical analysis of Figure 3I.

Number of families	1					
Number of comparisons per family	21					
Alpha	0.05					
Tukey's multiple comparisons test	Mean Diff.	95.00% CI of diff.	Below threshold?	Summary	Adjusted P Value	
A2-BL3 vs. A2-BL3-GP (Bulk)	-1.563	-1.796 to -1.329	Yes	****	<0.0001	A-B
A2-BL3 vs. A2-BL3-GP5	-1.607	-1.827 to -1.386	Yes	****	<0.0001	A-C
A2-BL3 vs. A2-BL3-GP8	-1.198	-1.419 to -0.9781	Yes	****	<0.0001	A-D
A2-BL3 vs. A2-BL3-GP10	-1.127	-1.347 to -0.9065	Yes	****	<0.0001	A-E
A2-BL3 vs. A2-BL3-GP18	-1.510	-1.730 to -1.290	Yes	****	<0.0001	A-F
A2-BL3 vs. A2-BL3-GP19	-3.242	-3.462 to -3.021	Yes	****	<0.0001	A-G
A2-BL3-GP (Bulk) vs. A2-BL3-GP5	-0.04417	-0.2183 to 0.1299	No	ns	0.9828	B-C
A2-BL3-GP (Bulk) vs. A2-BL3-GP8	0.3642	0.1901 to 0.5383	Yes	****	<0.0001	B-D
A2-BL3-GP (Bulk) vs. A2-BL3-GP10	0.4358	0.2617 to 0.6099	Yes	****	<0.0001	B-E
A2-BL3-GP (Bulk) vs. A2-BL3-GP18	0.05250	-0.1216 to 0.2266	No	ns	0.9598	B-F
A2-BL3-GP (Bulk) vs. A2-BL3-GP19	-1.679	-1.853 to -1.505	Yes	****	<0.0001	B-G
A2-BL3-GP5 vs. A2-BL3-GP8	0.4083	0.2526 to 0.5640	Yes	****	<0.0001	C-D
A2-BL3-GP5 vs. A2-BL3-GP10	0.4800	0.3243 to 0.6357	Yes	****	<0.0001	C-E
A2-BL3-GP5 vs. A2-BL3-GP18	0.09667	-0.05905 to 0.2524	No	ns	0.4573	C-F
A2-BL3-GP5 vs. A2-BL3-GP19	-1.635	-1.791 to -1.479	Yes	****	<0.0001	C-G
A2-BL3-GP8 vs. A2-BL3-GP10	0.07167	-0.08405 to 0.2274	No	ns	0.7671	D-E
A2-BL3-GP8 vs. A2-BL3-GP18	-0.3117	-0.4674 to -0.1560	Yes	****	<0.0001	D-F
A2-BL3-GP8 vs. A2-BL3-GP19	-2.043	-2.199 to -1.888	Yes	****	<0.0001	D-G
A2-BL3-GP10 vs. A2-BL3-GP18	-0.3833	-0.5390 to -0.2276	Yes	****	<0.0001	E-F
A2-BL3-GP10 vs. A2-BL3-GP19	-2.115	-2.271 to -1.959	Yes	****	<0.0001	E-G
A2-BL3-GP18 vs. A2-BL3-GP19	-1.732	-1.887 to -1.576	Yes	****	<0.0001	F-G

Supplemental Figures

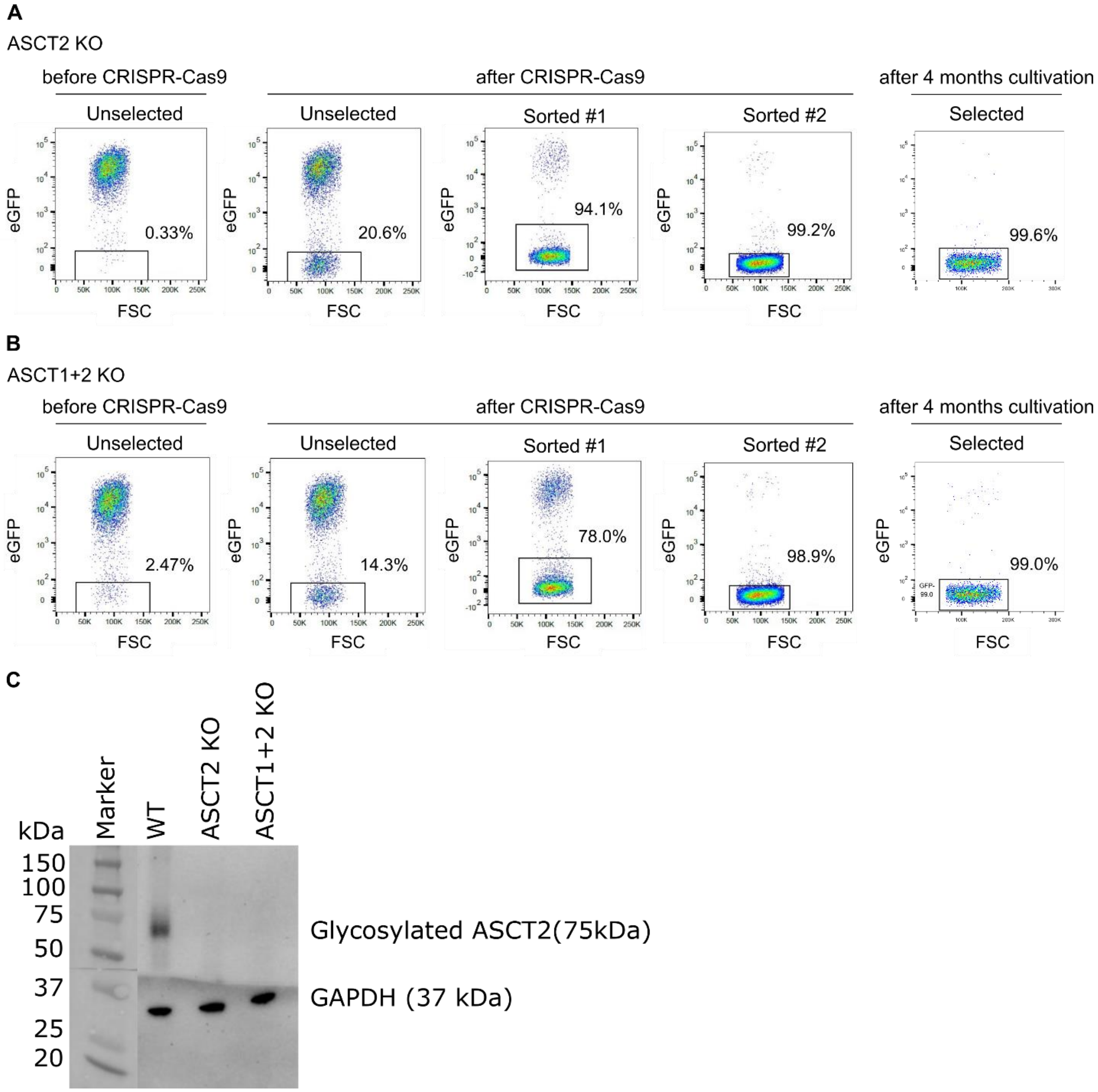

Figure S1. Generation of ASCT2 and ASCT1+2 knockout (KO) HEK293T cell lines. **A-B.** Selection of ASCT2 KO cells (A) and ASCT1+2 KO cells (B) via cell sorting. Selected cells maintained their loss to infectivity after cultivating the cell lines for over four months. **C.** Western blotting of ASCT2 confirmed absence of ASCT2 protein on both ASCT2 KO and ASCT1+2 KO HEK293T packaging cell lines.

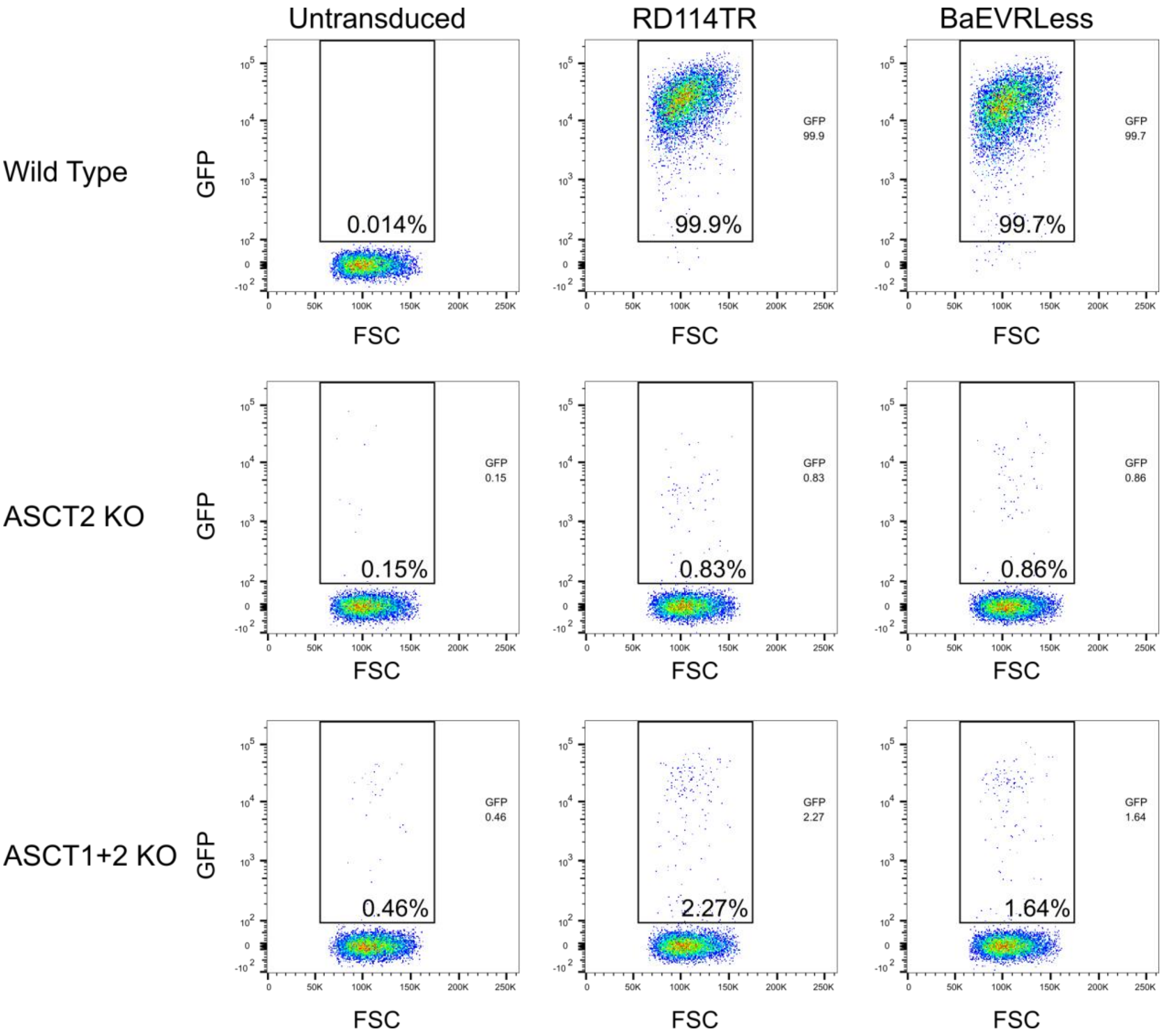

Figure S2. Transduction of wild type, ASCT2 KO and ASCT1+2 KO cells with RD114TR or BaEVRLess pseudotyped alpha-retroviral particles.

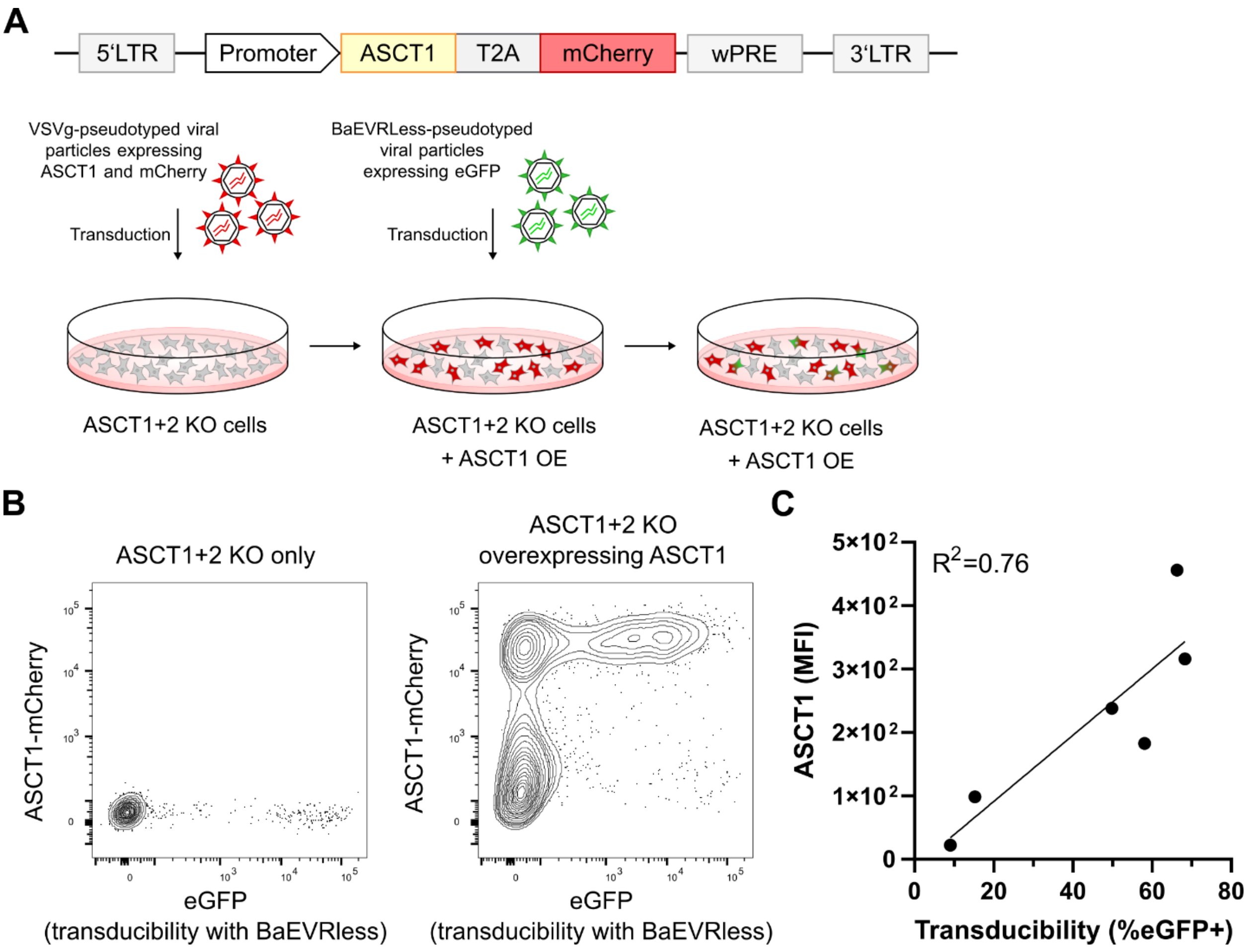

Figure S3. Evaluation of ASCT1 as a cell entry receptor for BaEVRLess pseudotyped viral particles. **A.** Schematic of the lentiviral vector used to overexpress ASCT1 followed by mCherry as a fluorescent reporter. Workflow of overexpressing ASCT1 (ASCT1 OE) on ASCT1+2 KO cells and testing their transducibility towards BaEVRLess pseudotyped alpha-retroviral vectors. **B.** Flow cytometry plot showing transducibility of ASCT1 overexpressing cells (mCherry-positive, right panel) cells with BaEVRLess pseudotyped retroviral particles expressing eGFP compared to ASCT1+2 KO cells only (left panel). **C.** Correlation of ASCT1 expression levels based on mean fluorescence intensity (MFI) to the transducibility of the cells with BaEVRLess pseudotyped retroviral vectors expressing eGFP.

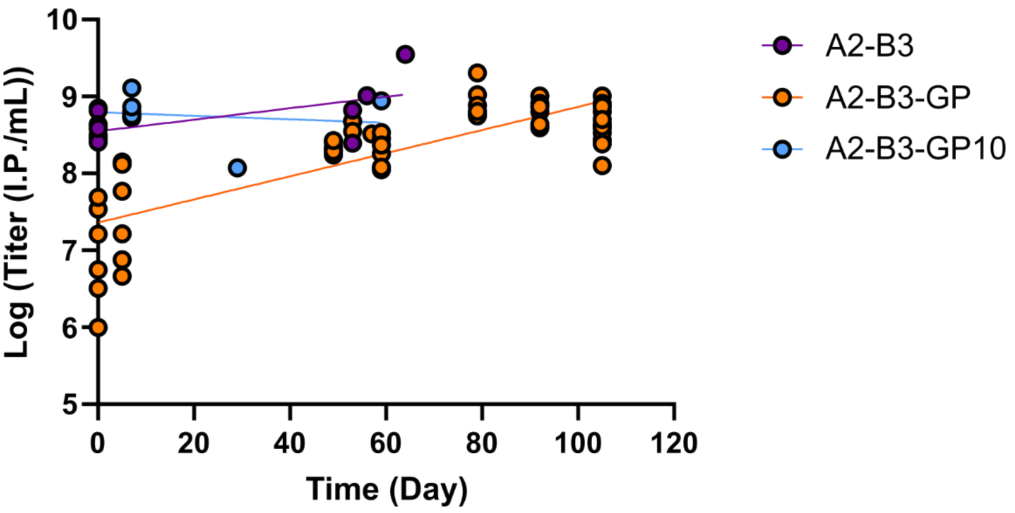

Figure S4. Analysis of the stability of viral titers from different packaging cell lines over time.

A

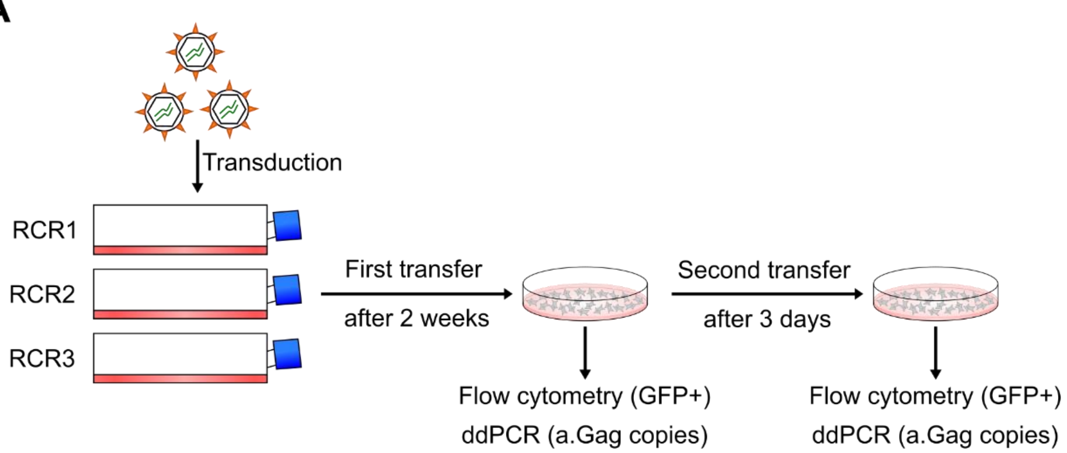

B

Sample	1st transfer (GFP+ of viable cells)	1st transfer (a.Gag of total droplets)	2nd transfer (GFP+ of viable cells)	2nd transfer (a.Gag of total droplets)
RCR 1	0 of 1450000	0 of 15853 droplets<br>0 of 15876 droplets<br>1 of 14014 droplets	0 of 583000	0 of 13066 droplets<br>0 of 12484 droplets<br>0 of 13821 droplets
RCR 2	0 of 1420000	0 of 16569 droplets<br>0 of 15731 droplets<br>0 of 16482 droplets	0 of 607000	0 of 15868 droplets<br>0 of 16559 droplets<br>0 of 14703 droplets
RCR 3	0 of 1460000	0 of 15714 droplets<br>0 of 15137 droplets<br>0 of 16332 droplets	0 of 637000	0 of 15399 droplets<br>0 of 13747 droplets<br>0 of 14732 droplets
UTD	0 of 1170000	0 of 16830 droplets<br>0 of 14879 droplets<br>0 of 15354 droplets	0 of 234000	0 of 16475 droplets<br>0 of 17042 droplets<br>0 of 15376 droplets

Figure S5. Testing for replication-competent retroviral particles. **A.** Schematic showing the workflow of testing for RCR particles. **B.** Results of the testing for alpha-retroviral RCR particles in the stable BaEVRLess alpha-retroviral packaging cell line. UTD=untransduced.
